# Age-related changes in cerebellar and hypothalamic function accompany non-microglial immune gene expression, altered synapse organization, and excitatory amino acid neurotransmission deficits

**DOI:** 10.18632/aging.101040

**Published:** 2016-09-20

**Authors:** Stephen J. Bonasera, Jyothi Arikkath, Michael D. Boska, Tammy R. Chaudoin, Nicholas W. DeKorver, Evan H. Goulding, Traci A. Hoke, Vahid Mojtahedzedah, Crystal D. Reyelts, Balasrinivasa Sajja, A. Katrin Schenk, Laurence H. Tecott, Tiffany A. Volden

**Affiliations:** ^1^ Division of Geriatrics, University of Nebraska Medical Center, Durham Research Center II, Omaha, NE 68198, USA; ^2^ Monroe-Meyer Institute, University of Nebraska Medical Center, Durham Research Center II, Omaha, NE 68198, USA; ^3^ Department of Radiology, University of Nebraska Medical Center, College of Medicine, Omaha, NE 68198, USA; ^4^ Department of Psychiatry and Behavioral Sciences, Northwestern University, Chicago, IL 60611, USA; ^5^ The Institute for Addiction Sciences and Psychology (IRSA), Tehran, Iran; ^6^ Department of Physics, Randolph College, Lynchburg, VA 24503, USA; ^7^ Department of Psychiatry, University of California, San Francisco, San Francisco, CA, 94158, USA

**Keywords:** aging, pattern recognition receptor (PRR), hypothalamus, cerebellum, feeding, mouse physical activity, microarray

## Abstract

We describe age-related molecular and neuronal changes that disrupt mobility or energy balance based on brain region and genetic background. Compared to young mice, aged C57BL/6 mice exhibit marked locomotor (but not energy balance) impairments. In contrast, aged BALB mice exhibit marked energy balance (but not locomotor) impairments. Age-related changes in cerebellar or hypothalamic gene expression accompany these phenotypes. Aging evokes upregulation of immune pattern recognition receptors and cell adhesion molecules. However, these changes do not localize to microglia, the major CNS immunocyte. Consistent with a neuronal role, there is a marked age-related increase in excitatory synapses over the cerebellum and hypothalamus. Functional imaging of these regions is consistent with age-related synaptic impairments. These studies suggest that aging reactivates a developmental program employed during embryogenesis where immune molecules guide synapse formation and pruning. Renewed activity in this program may disrupt excitatory neurotransmission, causing significant behavioral deficits.

## INTRODUCTION

Aging individuals vary in their susceptibility for developing mobility impairment or involuntary weight loss. For example, mobility impairments were noted in 36% of subjects enrolled in a large 6 year longitudinal trial studying adults 65+ years old who were functionally intact at enrollment [1]. Much of this loss could not be attributed to sarcopenia, Parkinson's disease, or stroke. Similarly, about 15% of adults experience involuntary age-related weight loss in the absence of cancer, gastrointestinal disease, or stroke [2].

Weight loss and mobility declines are both harbingers of frailty, a clinical syndrome that increases the risk of functional loss and death [3]. Although etiologies of age-related mobility and energy balance impairments are not well understood, there is good reason to suspect that changes in CNS structure and function may be involved. CNS aging alters brain anatomy [4,5]. Progressive loss or altered function of synapses in a specific CNS region ultimately impairs behaviors under that structure's control [6]. How aging changes synapse organization and overall synaptic activity, ultimately causing functional impairment, remains poorly understood. These questions have widespread societal impact, since considerable spending will be required in the coming decade to manage problems directly attributable to CNS aging.

We focused on determining the CNS processes underlying two clinically-significant phenotypes associated with aging: decreased physical activity and weight loss. We first demonstrate that different mouse strains have varying susceptibilities to age-related impairments in mobility and body weight. We then examine the regional impact of age-related changes in gene expression within the cerebellum and hypo-thalamus, two key regions that organize activity and energy-balance behaviors, and translate these findings to the human cerebellum. Finally, we studied the effect of these age-related changes in gene expression on synapse organization, and overall excitatory synaptic activity within these two CNS regions.

## RESULTS

### Aging mice have functional deficits

**Figure 1:**
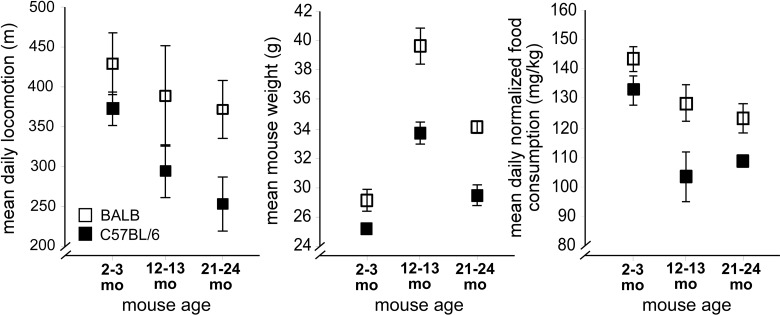
Strain-specific impairments in mouse mobility and energy balance occur with aging Left: Decreased mean daily locomotion in aged C57BL/6 mice (filled rectangles), with preserved mean daily locomotion in aged BALB mice (open rectangles). Center: Body weight in aged C57BL/6 and BALB mice decreases between 12-13 months and 21-24 months. Right: Normalized food intake. For all figures, error bars are ± 1 standard error of the mean.

We began by examining how aging affected mouse functional status: food intake, water intake, and physical activity. These behaviors were evaluated for 13-18 days in a custom-designed home cage behavioral monitoring (HCM) system following 5 days of acclimation [7]. We tested cohorts of young (2-3 mo), middle-aged (12-13 mo), and aged (21-24 mo) BALB and C57BL/6 mice. These two strains have distinct breeding lineages [8], and thus provide an indication of how genetic diversity might influence age-related CNS phenotypes. In C57BL/6 mice, locomotion (Figure 1, left panel) markedly decreased with age (*p*<0.011, F_2,30_=5.2, one-way ANOVA), with a 32% difference noted between young and aged mice by *post hoc* testing (*p*<0.013). By contrast, BALB mice demonstrated no age-related locomotor decreases. Furthermore, false discovery rate analysis (Supplemental Table 1A) showed that aged C57BL/6 mice were more likely to have mobility impairments when compared to either young C57BL/6 or aged BALB mice. For example, aged C57BL/6 mice had more impairments in locomotor-associated behaviors when compared to young C57BL/6 mice (*p*<0.042, χ^2^ test) or when compared to aged BALB mice (*p*<0.026). Similarly, aged C57BL/6 mice had more impairments of nonlocomotor movement-associated behaviors when compared to young C57BL/6 mice (*p*<0.046) or when compared to aged BALB mice (*p*<0.05).

**Figure 2:**
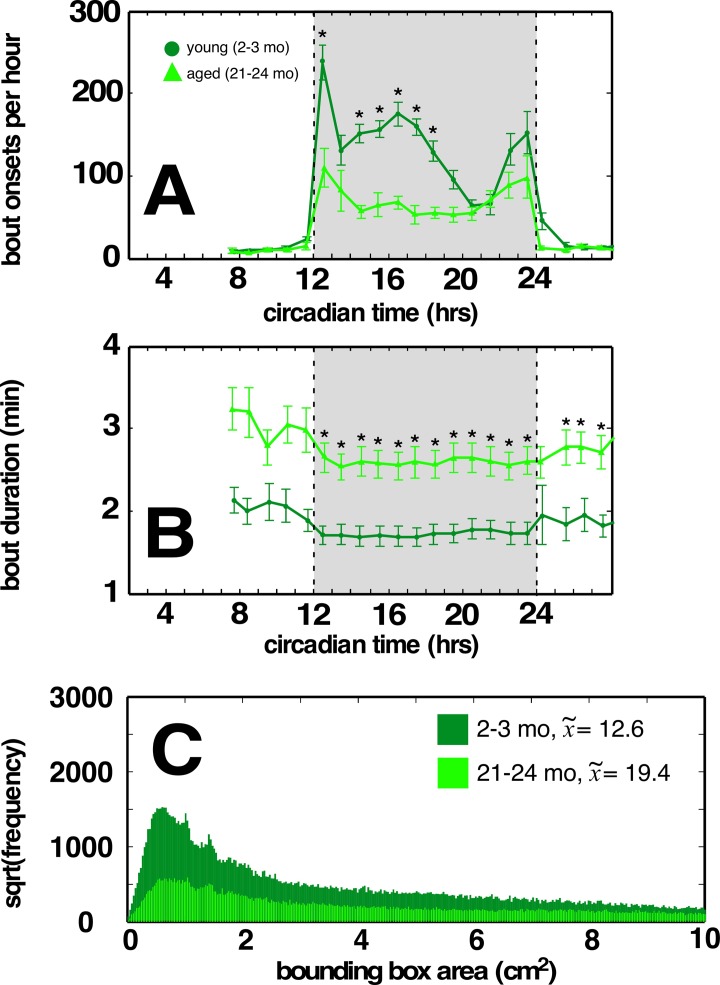
Aged C57BL/6 mice have fewer locomotor bouts during the dark cycle, and a greater proportion of weaving locomotor bouts compared to young cohorts (**A**) Aged C57BL/6 mice display fewer bouts of dark cycle locomotion compared to young cohort. (**B**) Increased locomotor bout durations in aged C57BL/6 mice. (**C**) Distribution of minimum bounding rectangle areas (MBRs; cut off at 10 to better show small rectangle areas) for locomotor bouts of young and aged C57BL/6 mice. Smaller MBRs indicate more direct locomotor paths. Median values for each cohort in legend. Traces in light green correspond to aged mice, dark green correspond to young mice. Greyed region depicts dark cycle, dashed lines indicate dark cycle onset and offset, respectively. Asterisks indicate *p*<0.01, Bonferroni corrected; error bars are ± 1 standard error of the mean.

Further dissection of this mobility deficit revealed a significant decrease in the overall number of locomotor bouts occurring during the circadian dark cycle as well as decreased locomotor speed (Figure 2A). This de-crease in locomotor bout frequency was incompletely compensated by an increase in locomotor bout duration (Figure 2B). Similar changes are observed across aging human cohorts [9]. Furthermore, locomotor paths of young C57BL/6 mice (Figure 2C) were significantly straighter (*p*<<0.001; across-mouse variability *p*<<0.001, within-mouse variability no effect) compared to locomotor paths of aged C57BL/6 mice, suggesting an age-related impact on cerebellar function (and related to tandem gait impairment commonly seen in older adults). By contrast, we observed no age-related difference in overall locomotion, locomotor bouts, or locomotor path straightness in BALB mice (Supplemental Figure 1A, B respectively, green traces).

**Figure 3:**
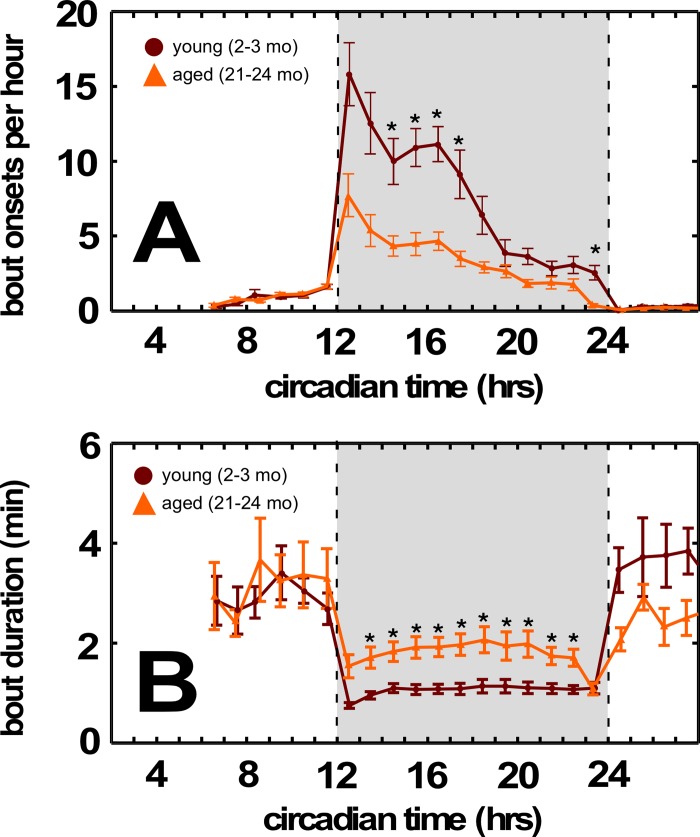
Aged BALB mice have fewer feeding bouts during the dark cycle (**A**) Decreased feeding bouts in aged BALB mice. (**B**) Increased feeding bout duration in aged BALB mice. Traces in light orange correspond to young mice; traces in dark orange correspond to aged mice. Grayed region depicts dark cycle, dashed lines indicate dark cycle onset and offset, respectively. Asterisks indicate *p*<0.01, Bonferroni corrected; error bars are ± 1 standard error of the mean.

By contrast, aging BALB mice demonstrated dysregulated overall energy balance. Normal aging is characterized by body weight maintenance, and decreased basal metabolic rate, activity, and food consumption. Both BALB and C57BL/6 mice show significant weight loss between the middle-aged and aged cohorts (Figure 1, center panel, *p*<0.05, *p*<0.0002 respectively, one sided t-test). However, we found no significant differences between middle-aged and aged BALB mice in peak oxygen consumption ($\dot{V}O_{2}$), carbon dioxide production ($\dot{V}O_{2}$), or adiposity (Supplemental Figure 2A). Aged BALB mice also maintain activity levels similar to those of young BALB mice, while aged C57BL/6 mice significantly decrease their activity. Furthermore, aged BALB mice tend to consume less chow (normalized to body weight) compared to middle-aged BALB mice, while aged C57BL/6 mice tend to consume more chow compared to middle-aged C57BL/6 mice (Figure 1, right panel). False discovery rate analysis further demonstrated that aged BALB mice showed impairments in more feeding-associated behaviors compared to aged C57BL/6 mice (*p*<0.036; Supplemental Table 1B). Further analysis of energy balance dysregulation in aged BALB mice revealed a significant decrease in the overall number of circadian dark cycle feeding bouts and decreased bout food intake (Figure 3A). Again, this decrease in feeding bouts was incompletely compensated by an increase in feeding bout duration, leading to a 10-15% decrease in overall feeding in aged BALB mice (compared to young cohort, Figure 3B). In C57BL/6 mice, no age-related difference in overall food intake or feeding bouts was appreciated (Supplemental Figure 2B). Age-related changes in feeding patterns similar to aged BALB mice have also been reported in older human populations [10].

### Altered CNS immune gene expression in aged mice

The molecular events underlying the above-described functional impairments remain poorly understood. To obtain an unbiased view of mRNA transcriptional changes associated with these behavioral deficits, we used whole mouse genome microarrays. Whole tissue cerebellar and hypothalamic mRNA expression was determined and compared between cohorts of young, middle-aged, and aged BALB and C57BL/6 mice (per Methods). Transcripts obtained originate from neuronal soma and dendrites, with minimal contribution from axonal projections [11]. Signal strength characteristics across all chips were similar, as were cross-sample correlations for each of the individual groups, suggesting good technical microarray performance (Supplemental Figure 3A-D for BALB hypothalamus, BALB cerebellum, C57BL/6 hypothalamus, and C57BL/6 cerebellum, respectively).

**Figure 4:**
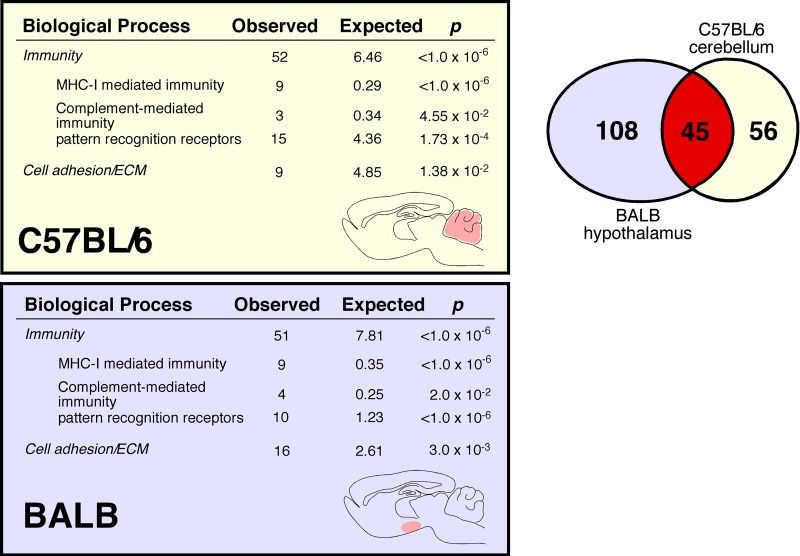
Age-associated increase in C57BL/6 cerebellar and BALB hypothalamic expression of immune transcripts Venn diagram demonstrates that although these two regions have different cellular architectures and functions, they share considerable overlap in age-related changes in gene expression. In male C57BL/6 cerebellum, we identify 101 differentially expressed genes (DEGs), 100 upregulated in aged mice. In male BALB hypothalamus, we identify 153 DEGs, 113 upregulated in aged mice. Of note, 45 of these genes are differentially expressed in both C57BL/6 cerebellum and BALB hypothalamus; the probability of this occurring by chance is *p*<<0.0001. We measured gene expression with Agilent Whole Mouse Genome 4x44k arrays that use 60-mer probes to detect 41,174 full length mouse genes and ESTs. Following array quality control and normalization, we identified differentially expressed genes by log posterior odds (B) values > 0 (Supplemental Methods).

**Figure 5:**
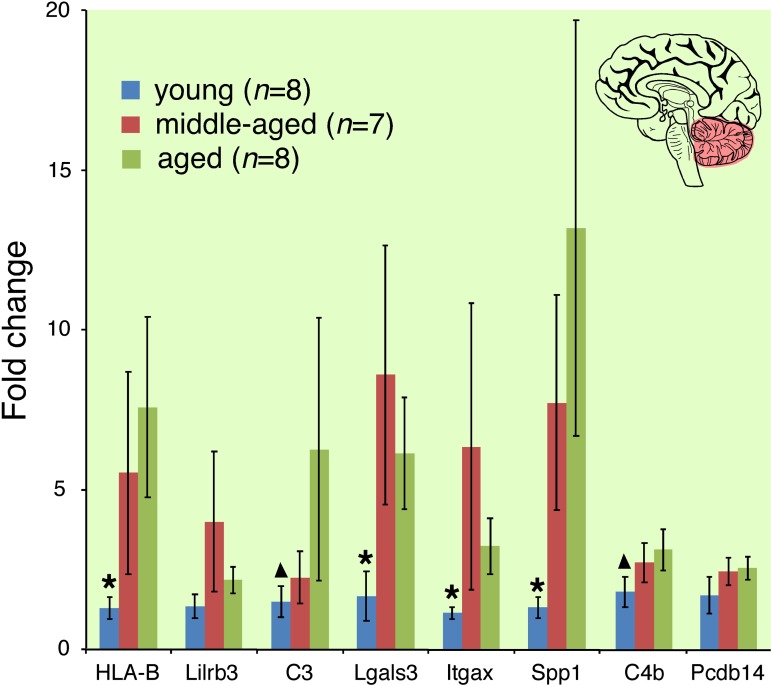
Cerebellar immune transcript expression increases with age in community-dwelling adults Asterisk indicates gene expression in young cohort significantly different from middle-aged and aged cohorts (*p*<0.05); triangle indicates gene expression in young cohort significantly different from aged cohort. Analogous genes were differentially expressed in the cerebellum of aged C57BL/6 mice with motor deficits.

Aging evokes modest changes in overall CNS gene expression (Figure 4, Venn diagram). Lists of genes differentially expressed between young and aged mice are provided in Supplemental Table 2 (BALB hypothalamus, BALB cerebellum, C57BL/6 hypothalamus, C57BL/6 cerebellum respectively). These changes occurred in both a strain- and site-specific manner (Figure 4 comparing BALB hypotha-lamus and C57BL/6 cerebellum; Supplemental Figure 4 comparing BALB cerebellum and C57BL/6 hypo-thalamus). In whole cerebellar and hypothalamic tissue of C57BL/6 and BALB mice, respectively, there was marked overexpression of transcripts characterized by ontology as having immune/defense function (*p*<<0.001 for both regions; Figure 4, also see Supplemental Table 2). Ontology analysis suggested that these transcripts belonged to specific functional categories, including classical major histocompatibility complex I (*e.g*. H2-D1, H2-K1), atypical major histocompatibility complex I (*e.g*. H2-Q1, H2-Q7), complement (*e.g*. C3, C1q, C4b), cytokine/chemokine (*e.g*. Ccl6, Ccl12), pattern recognition receptors (*e.g*. Tlr2, Clec7a, Lgals3, Trem2, F_c_Rs, Lilrb3), and cell adhesion molecules (*e.g*. Itgax, Lyz, Timp1). Predicted protein-protein interactions (STRING 9.1; string-db.org) further suggest that many genes overexpressed in the C57BL/6 cerebellum and BALB hypothalamus form membrane-bound signaling complexes (*e.g*. Fcgr3-Fcer1g-Tyrobp-Lilrb3-Ms4a6d-C1q; Clec7a-Tyrobp-Emr1-Ctss-Ly86-Igsf6-Mpeg1; Lgals3-Cd68-Capg-S100a4-Anxa4-Lgals3bp) that con-verge to activate NFκB. RT-qPCR confirmed these age-related changes in gene expression for selected loci in whole hypothalamic tissue (Supplemental Figure 5). 16% of the cerebellar genes differentially expressed between young and old C57BL/6 mice are known NFκB targets [12]; this value rises to 33% by including NFκB target predictions by TRANSFAC (ver 7.0 Public 2005) [13]. Similarly, 10% of the hypothalamic genes differentially expressed between young and old BALB mice are known NFκB targets, rising to 23% by including TRANSFAC predictions. Intriguingly, in human cerebellum we find age-related increases in expression of mRNAs homologous to those identified in the C57BL/6 mouse cerebellum (Figure 5). These changes occurred across all of the above categories, including MHC I molecules (HLA-B), complement (C3, C4b), PRRs (Lilrb3, Lgals3) and CAMs (Itgax, Spp1).

### Immune genes do not localize to microglia

**Figure 6:**
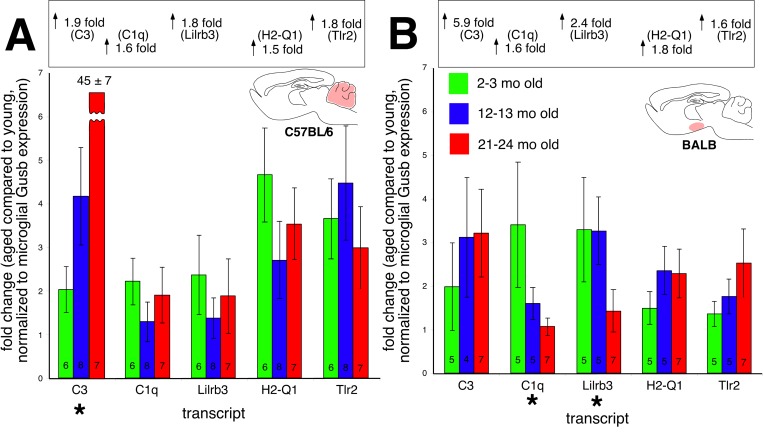
Microglial-specific expression of immune transcripts found upregulated with age in whole tissue (**A**) C57BL/6 cerebellum and (**B**) BALB hypothalamus. Values in each bar depict number of biological replicates. With the exception of C3 expression in the C57BL/6 cerebellum, no transcripts increase microglial expression with age. Values in the boxes above each graph show overall changes in transcript expression from whole tissue RT-qPCR experiments.

Age-related increases in the CNS expression of immune/defense related transcripts have been attributed to neuroinflammation [14-16]. If this were the case, then a majority of these changes should localize to microglia. Microglia are resident CNS mononuclear phagocytes, and account for the vast majority of CNS immunocytes. Furthermore, microglial activation is a key and necessary step supporting neuroinflammation. We used a multi-step magnetic bead based separation process combined with molecular and functional validations [17] to obtain highly enriched populations (65-90% CD11b(+) total cells within suspension) of both parenchymal and perivascular microglia from the cerebellum and hypothalamus of young, middle-aged, and aged BALB and C57BL/6 mice. RT-qPCR gene expression assays found that these microglia clearly did not contribute to most of the observed age-related changes in whole tissue gene expression (Figure 6). Microglia only contributed to the age-associated C3 increase in the C57BL/6 cerebellum (Figure 6A). In the BALB hypothalamus (Figure 6B), expression of C1q and Lilrb3 were decreased in microglia derived from aged mice (*p*<0.01). These results strongly suggest that most of the age-related changes in cerebellar and hypothalamic immune/defense gene expression do not localize to CNS microglia.

### Synapse changes accompany altered PRR expression

**Figure 7:**
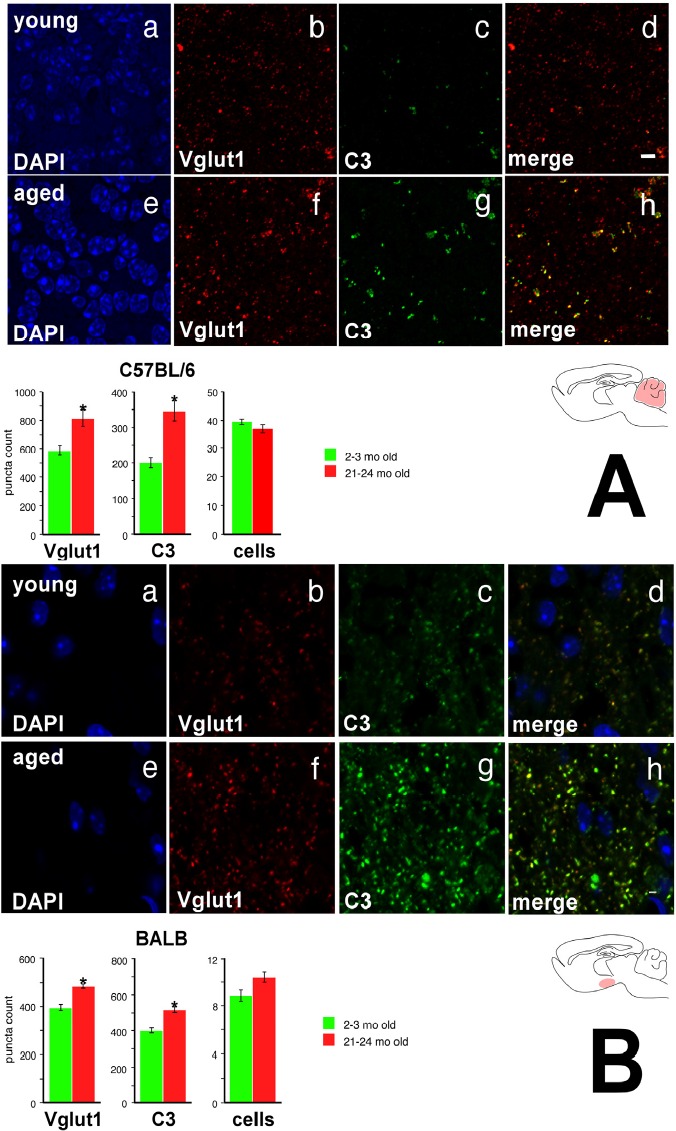
Vglut1 and C3 show increased expression in the cerebellar internal granule cell layer of the aged C57BL/6 mouse and in the hypothalamic arcuate nucleus of the aged BALB mouse (**A**) C57BL/6 cerebellar internal granule cell layer. **a** DAPI stain, young. **b** Vglut1 immunoreactivity, young. **c**. C3 immunoreactivity, young. **d** Merge, young. Note significant colocalization of Vglut1 and C3 staining, particularly for more intense puncta. **e** DAPI, aged. **f** Vglut1, aged. **g** C3, aged. **h** Merge, aged. Bottom: Quantification of Vglut1, C3, and DAPI. (**B**) BALB hypothalamic arcuate nucleus. Panels **a**-**h** as above. Again, note significant colocalization of Vglut1 and C3 staining, particularly for more intense puncta. Asterisk denotes *p*<0.01. Scale bar 4 μm.

In this light it is interesting to note that recent studies have found that pattern recognition molecules play an important role during both synaptogenesis and synaptic pruning phases of CNS development [18]. In the developing mouse thalamus, MHC Class I molecules demonstrate an activity-dependent expression pattern [19], and loss of these molecules leads to decreased synaptic pruning [20] and altered visual cortex plasticity [21]. Similar disruptions of synaptic pruning or plasticity are observed with loss of C1q (in the thalamus), PirB (in the primary visual cortex), and H2-K^b^/H2-D^b^ (in the cerebellar Purkinje layer) [22-24]. MHC Class I molecules also regulate hippocampal synaptic activity [25]. We thus tested the hypothesis that increased expression of these PRR transcripts was associated with altered excitatory synapse organization. Given our earlier findings, we predicted that age-related synaptic changes should be more pronounced in the cerebellum of C57BL/6 mice compared with BALB mice, and in the hypothalamus of BALB mice compared with C57BL/6 mice. To measure synaptic counts in these regions at highest accuracy, we employed a modified array tomography protocol. Ultrathin (90 nm) sections were prepared from the cerebellum of BALB and C57BL/6 mice from young and aged cohorts; thin (10 μm) sections were prepared from the hypothalamus of BALB and C57BL/6 mice from young and aged cohorts (Supplemental Methods). Cell nuclei were visualized with DAPI, and excitatory synapses were visualized with antibodies directed against Vglut1. We chose C3 as a molecule increased in expression in both BALB hypothalamus and C57BL/6 cerebellum. Figure 7A shows representative photomicrographs in young and old C57BL/6 cerebellum, as well as quantified puncta counts (quantification workflow provided in Supplemental Figure 6); Figure 7B shows the equivalent figure for the young and old BALB hypothalamic arcuate nucleus.

In the cerebellar internal granule cell layer, we observe a marked age-associated increase in both Vglut1 and C3 expression in C57BL/6, but not BALB, mice (Figure 7A; 2-way ANOVA with mouse strain and age as primary factors; strain × age interaction *p*<0.04 for Vglut1; *p*<0.001 for C3). Specifically, in C57BL/6 mice, internal granule cell layer Vglut1 expression increases nearly 20% with age; in BALB mice, Vglut1 baseline expression is lower in young mice, and does not significantly change with age. We did not find any size difference in counted puncta. No significant differences were appreciated in either Vglut1 or C3 expression in aged BALB mice compared to young BALB counterparts (Supplemental Figure 7A). There were no age-related differences noted in overall cell count in either C57BL/6 or BALB mice, making neuro-degeneration an unlikely explanation for this phenotype.

We further note that in the hypothalamic arcuate nucleus, we observe a marked age-associated increase in both Vglut1 and C3 expression in BALB (Figure 7B), but not C57BL/6 (Supplemental Figure 7B), mice (2-way ANOVA with mouse strain and age as primary factors; strain × age interaction *p*<0.001 for Vglut1; *p*<0.007 for C3). While young BALB and C57BL/6 mice both show the same degree of Vglut1 expression, with aging we find a nearly 20% increase in BALB arcuate Vglut1 expression that is not seen in the aged C57BL/6 mouse. Again, no size difference was noted in counted puncta, and there were no age-related differences in arcuate hypothalamus cell counts. These findings suggest that aging increases the number of excitatory synapse components in CNS regions where we observe increased expression of immune, complement, and pattern recognition genes with age, but does not change excitatory synapse organization in CNS regions where these age-related patterns of gene expression do not occur.

### Functional impairment in aged synapses

**Figure 8:**
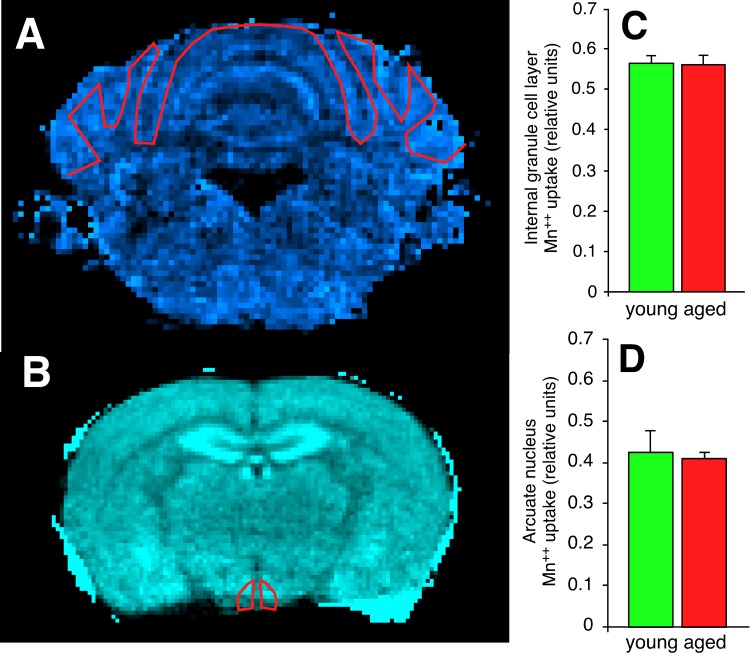
Synapses in the cerebellar internal granule cell layer of the aged C57BL/6 mouse and in the hypothalamic arcuate nucleus of the aged BALB mouse have functional deficits in excitatory amino acid neurotransmission (**A**) Scaled difference MRI image of mouse cerebellum at approximately bregma −6.96 mm. Regions with higher Ca^++^ uptake are brighter on this colormap. The red line depicts the region-of-interest (ROI) drawn to include the internal granule cell layer. (**B**) Scaled difference MRI image of mouse hypothalamus at approximately bregma −1.46 mm. (**C**, **D**) Despite both regions demonstrating increased expression of regional-appropriate vesicular glutamate transporters, there is no evidence of increased post-synaptic Ca^++^ uptake in either the cerebellar internal granule cell layer (**C**) or the hypothalamic arcuate nucleus (**D**).

To determine if the increased synapse counts observed in the aged C57BL/6 cerebellum and aged BALB hypothalamus were associated with a functional outcome, we assessed excitatory amino acid activity across the entire cerebellar internal granule cell layer and hypothalamus using manganese-enhanced MRI (MEMRI, supplemental methods). Neuronal Mn^++^ transport occurs in an activity-dependent manner analogous to calcium; however, Mn^++^ is paramagnetic, causing reduction of T_1_ in proportion to concentration, which can be measured using T_1_ weighted imaging. Thus, Mn^++^ enhances MRI tissue signals in direct proportion to overall excitatory amino acid activity. Our results show no age-related increase in either C57BL/6 cerebellar internal granule cell layer (Figure 8A,C) or BALB hypothalamic arcuate nucleus (Figure 8B,D) excitatory amino acid activity. These findings do not rule out the possibility of increased excitatory amino acid activity below the MEMRI detection threshold in aged compared to young mice; however, changes of this magnitude are below expected biological signaling thresholds, and would require electrophysiology to verify. To rule out the possibility that age-related vascular disease contributed to our phenotypes, we further evaluated diffusion tensor images for all subjects. There were no significant age-related changes in water diffusion characteristics or vascular disease in either C57BL/6 or BALB mice (Supplemental Figure 8). These findings support the conclusion that the increased number of excitatory synapses present in the aged C57BL/6 cerebellar internal granule cell layer and aged BALB arcuate hypothalamic nucleus have a currently uncharacterized functional deficit.

## DISCUSSION

In summary, we have framed a new model that further clarifies how cerebellar and hypothalamic aging cause mobility and energy balance impairments in aging mice. We demonstrate patterns of dysregulated CNS function associated with age-related behavioral phenotypes at molecular, cellular, and organ scales of organization. We further show that these patterns of dysregulated function have strong genetic predispositions, with some mouse strains developing specific age-related impairments in mobility or feeding, while other mouse strains do not. Analogous situations are observed in human aging [26, 27]. We show that aging reactivates CNS regional expression of classical and atypical MHC-1, complement, PRRs, and CAMs in a nonmicroglial context. Similar findings have been observed regarding C1q, whose expression increases in the aging mouse hippocampus and is accompanied by synaptic and behavioral impairments [28]. We also observe increased cerebellar expression of homologous transcripts in older human subjects. At a synaptic level of organization, we observe more excitatory presynaptic puncta in CNS regions where age-associated increases in immune gene expression occur. Similar age-related synapse dynamics have been noted in primary sensorimotor cortex of aged mice [29]. This increased number of excitatory synaptic components in both the aged cerebellum and hypothalamus did not evoke increased postsynaptic Ca^++^, suggesting an age-associated synaptic defect. This defect may arise from either altered presynaptic vesicular glutamate content [30, 31], decreased synaptic vesicle exocytosis probability [32, 33], or attenuated postsynaptic Ca^++^ signaling (multiple potential mechanisms affecting AMPAR trafficking and postsynaptic persistence). Ultimately, these deficits lead to impaired cerebellar function in C57BL/6 mice (demonstrated by their age-associated ataxia and locomotor loss) and impaired hypothalamic function in BALB mice (demonstrated by decreased food consumption in aged compared to middle-aged mice). Age-related losses in mobility and energy balance prominently affect substantial populations of older men and women, increasing health care utilization and costs while worsening personal quality of life. The concept that developmental programs active during embryogenesis, silent in adulthood, and reactivated with advancing age could lead to functional deficits is novel, and may be relevant to other organ systems.

We propose that age-related functional deficits arise through the following sequence. First, damage-associated molecular patterns (DAMPs) within the CNS matrix activate a multiplicity of neuronal PRRs, including C3R, C1qR, Trem2, Fcer1g, Fcgr2b, Fcgr3, Clec7a, Lgals3, Lilrb3, and so on. We suspect that signaling from these sources stimulates NFκB activity *in vivo*; for example, mice with mutations delaying IκBɑ (NFκB primary functional inhibitor) synthesis show increased NFκB activity after excitatory neuronal stimuli, and developed more excitatory synapses in *in vitro* neuronal culture [34]. Most of the PRRs we identified signal through pathways that drive NFκB activation [35-37]. Furthermore, many of the genes we identified as differentially expressed with aging and associated with functional impairments are demonstrated NFκB targets [12, 13]. Under baseline conditions, NFκB is known to regulate neuronal responses to excitatory neurotransmission [38-40], including induction of BDNF [41], Grm2 [42], Grin2A [43], and Grin [44]. PRR signaling thus confounds neuronal NFκB signaling evoked by AMPAR/NMDAR activity, falsely indicating increased excitatory neuronal activity. Increased transcription at NFκB loci alters coordinated expression of excitatory synapse components, leading to imbalances in proteins required to assemble excitatory synapses and the creation of excitatory synapses with functional impairments. These synapses, as well as excess protein trafficked to the synapse, in turn undergo accelerated turnover, thus increasing matrix DAMP concentrations [45]. Continued activity of this positive feedback loop ultimately degrades hypothalamic and cerebellar synaptic organization, leading to functional loss. In support of this concept, interventions inhibiting hypothalamic IKKβ or NFκB activity in aged animals ameliorate age-related feeding phenotypes [46].

## METHODS

Young (Y, 2-3 mo), middle-aged (M, 12-13 mo), and aged (A, 21-24 mo) male C57BL/6 and BALB mice were obtained from the NIA aged rodent colony; human tissue gifts were obtained from the UNMC brain bank. Mouse behavioral studies were performed per [7]. Subject numbers for presented figures: BALB (Y *n*=10, M *n*=7, A *n*=11), C57BL/6 (Y *n*=10, M *n*=9, A *n*=11). Behavioral studies were replicated in at least two separate cohorts. Whole tissue hypothalamic and cerebellar RNA was purified by standard methods, assessed for degradation, and hybridized to Agilent 4x44k Whole Mouse Genome microarrays per manufacturer protocol. Array data analysis included quantile normalization, determination of differential gene expression, and classification of differential gene expression by ontology-based methods. Subject numbers for microarray studies were as follows: BALB hypothalamus (Y *n*=7, M *n*=5, A *n*=6); C57BL/6 cerebellum (Y *n*=4, M *n*=2, A *n*=6). Microglial studies were performed as described by [15]. Subject numbers were as follows: BALB hypothalamus (Y *n*=6, M *n*=8, A *n*=7); C57BL/6 cerebellum (Y *n*=5, M *n*=5, A *n*=7). For studies examining hypothalamic and cerebellar synapse organization, mice were sacrificed either by rapid decapitation (for cerebellar studies) or intracardiac perfusion (hypothalamic studies). Thin sections (90 nm cerebellum, 10 μm hypothalamus) were prepared and stained for nuclei, C3, and Vglut1. Multiple windows were visualized within the cerebellar internal granule cell layer and hypothalamic arcuate nucleus; cell counts were quantified by ImageJ and analyzed by ANOVA. Subject numbers were as follows: BALB hypothalamus (Y *n*=7, A *n*=7); C57BL/6 hypo-thalamus (Y *n*=4, A *n*=5); BALB cerebellum (Y *n*=3, A *n*=4); C57BL/6 cerebellum (Y *n*=4, A *n*=4). Mn^++^ enhanced MRI was performed per [47]. Subject numbers were as follows: BALB (Y *n*=5, A *n*=4); C57BL/6 (Y *n*=5, A *n*=5).

More Methods, MiQE, GEO archive accession number, UNMC digital link to the raw behavioral data could be found in Supplemental Material.

## SUPPLEMENTAL MATERIAL FIGURES


