# Supplementary material for: Age-related changes in cerebellar and hypothalamic function accompany non-microglial immune gene expression, altered synapse organization, and excitatory amino acid neurotransmission deficits
Source: Aging (Albany NY). 2016 Sep 20;8(9):2153–65. doi: 10.18632/aging.101040 (PMC5076456; doi:10.18632/aging.101040)
Supplement: Supplementary file 1 [file aging-08-2153-s001.pdf]

SUPPLEMENTAL MATERIAL

METHODS

Mouse husbandry

Mice were obtained from the NIA aged rodent colony. All animals were singly housed in UCSF- or UNMC-maintained animal housing for one to two weeks before being transferred to the behavioral monitoring facility; this allowed adequate time for animals to accommodate to the facility day-night cycle (7:00 AM lights on, 7:00 PM lights off). Mice with obvious medical problems (cataracts, skin lesions, tooth malocclusion, *etc.*) were excluded. Behavioral data collection was briefly (1.5-2 hrs) interrupted on a daily basis to measure animal food and water intake, and to provide fresh food (PicoLab Mouse diet 20 #5058) and water. Animal maintenance timing was staggered to prevent animals from synchronizing to the task. Temperature within the facility was maintained between 68 and 74°F; light-dark cycle was identical to that of the barrier facility. Mice not tested in the home cage monitoring system were acclimated to the housing facility for at least one week prior to use. For microarray, RT-qPCR, and microglial studies mice were sacrificed by decapitation followed by rapid brain dissection and placement of tissue into appropriate buffer or preparation of single cell suspensions. For array tomography experiments, mice were perfused by intracardiac administration of 0.1M phosphate buffered saline (PBS) followed by 0.4% paraformaldehyde (PFA) in 0.1M PBS. All studies fully complied with institutional and national guidelines for the ethical treatment of laboratory animals; all study protocols were approved through the UCSF and UNMC Institutional Animal Care and Use Committee (IACUC).

Home cage behavioral monitoring

Two mouse cohorts are presented: male BALB (2-3 mo old (Y), n=10; 12-13 mo old (M), n=7; 21-22 mo old (A), n=13), and male C57BL/6 (2-3 mo old, n=8; 23-24 mo old, n=8). Findings were confirmed in additional studies of Y, M, and A BALB (male and female), and C57BL/6 (male) cohorts. These sample sizes were arrived at through both prior experience and the limitation of 32 available home cage monitoring stations for use. All mice within a given strain-age cohort were biological replicates; our experimental design obviated the need for technical replicates. Mice were singly-placed in low profile cages (Allentown PC10196HT, 48×26×15 cm) modified by addition of a specially-designed feeding chamber, lickometer, and niche. Cage assignment was randomized using a Latin-squares design. All cohorts were habituated to the behavioral monitoring system for 5 days prior to study. Full details regarding the measurement and analytical capabilities of this system are beyond the scope of this text, but have been published [1, 2]. Briefly, the home cage monitoring system is capable of high spatial and temporal resolution of mouse locomotor, feeding, and drinking behavior. Each cage is balanced on a load-beam platform that can accurately determine mouse center-of-gravity to 1 cm spatial and 20 ms temporal resolution. Attempts to access an integrated food hopper are identified by the break pattern of an infrared photobeam. Mouse drinking patterns are identified by capacitance changes at the cage lick spout.

Home cage behavioral data quality control, classification, and analysis.

Investigators were not blinded to mouse cohort for these experiments; however, the automated nature of the behavioral quality control, classification, and analysis workflows minimized potential bias. Mouse behavioral data first underwent a brief user-guided quality control assessment to identify specific epochs (*e.g.* blocked photosensors, excessive position estimate drifts) or conditions (poor mouse health), where data may be unreliable. These epochs are removed from the dataset. Data loss from quality control is typically less than 1% of all data collected during an experiment. Automated behavioral classification algorithms then transform quality controlled sensor data to validated measures of feeding, drinking, and movement. Full explanations of these algorithms are available from Goulding and colleagues [1], and are briefly summarized here.

Feeding and drinking bouts are determined by modeling (for both the light and dark cycles) temporal and spatial properties of photobeam breaks and capacitive changes. Temporal properties are evaluated by creating a Gaussian mixture model of log-transformed inter-event interval durations (either photobeam or drinking) using estimation maximization (EM) methods. Spatial properties are evaluated by fitting bivariate Gaussian contours to the greatest distance achieved from the feeder or lick spout for each inter-event interval. Inter-event intervals contained within bivariate gaussians closest to the feeder or lick spout (fitted by kmeans) were assigned to occur at the respective device. A machine learning algorithm examines the gait speed and turning angle characteristics of all locomotor events not involving either the feeder or lickometer, and uses the distribution of these measures to determine if movements best conform to forward locomotion, movement in place, or no movement. We employ minimum bounding rectangles [3] (MBRs) to quantify

locomotor path weaving. Differences in MBRs were quantified by repeated measure ANOVA, with genotype as treatment factor, replicated for each animal over experimental days, and individual mice as covariates.

Following data classification and analysis, we examine family-wise error rate by false discovery [4], and quantify age-associated differences in 665 specific aspects of home cage behavior. To determine age-related differences over broad behavioral categories (feeding, drinking, locomotor, stopping, nonlocomotor movements), we employed an approach analogous to that used in comparing gene ontologies. We aggregated all measured behaviors into categories unambiguously associated with feeding, drinking, locomotion, stopping, and nonlocomotor movement. Measures that assessed two behaviors simultaneously (*e.g.* states containing both locomotion and activity) were included in both categories. We counted how many differential behaviors (criteria p<0.05 by t-test FDR) occurred in each category (*e.g.* our observed distribution). We generated an expected distribution by randomly selecting the same number of significant differential behaviors from the entire behavior list (without replacement, 10 permutation trials performed), and counting how many behaviors occurred in each category as above. A χ^2 statistic was then calculated from the observed and expected number of differentially expressed behaviors. All data analyses were performed in MATLAB (Mathworks, Natick, MA).

Metabolic assays

Dual energy X-ray absorptiometry (DEXA) studies were performed by standard protocol (modified from mutant mouse phenotyping center www.mmpc.org/shared/showFile.aspx?doctypeid=3&docid=104 and Whitelabs www.whitelabs.org/Lab%20Protocols/live%20animal%20protocols/DEXA%20scanning%20protocol.htm) using inhalational isoflurane anesthesia, a PIXImus scanner (GE Lunar, Inside/Outside Inc., WI), and Piximus 2.10 software. We chose the abdomen below the diaphragm (not including limbs or tail) as our region of interest for quantification. Our indirect calorimetry system (open circuit system; Oxymax Equal Flow, Columbus Instruments, OH) consisted of an air pump, CO_2 sensor (range 0%-0.8%; resolution 0.002% CO_2; drift <20 ppm CO_2/hr), paramagnetic O_2 sensor (range 0-100%; resolution 0.002% of specified range; drift <0.06% of specified range per 24 hrs), air dryer, controller, 8 hermetically sealed indirect calorimetry chambers (20.1×10.2×12.7 cm^3, part 760M-D8, Columbus Instruments), chamber photocell bracket (1.27 cm between photocells), photocell controller (Opto M3, Columbus Instruments), and software (Oxymax for Windows 4.49) running on dedicated hardware. Indirect calorimetry measures were taken per standard protocol (per MMPC, www.mmpc.org/shared/showFile.aspx?doctypeid=3&docid=194). Briefly, mice were fasted between 9:00 AM and 1:00 PM on the testing day. The system was turned on at 9:00 AM, and given 3 hrs to equilibrate before calibration. Calibration gases were 100% N_2 and a mixture of 0.5% CO_2/20% O_2/79.5% N_2 (span). Each station serially cycled through 5 min of data acquisition for the duration of data collection (1:00 PM to 5:00 PM). To determine basal metabolic rate, we averaged values obtained from the three epochs (15 min total) where each mouse demonstrated the least activity (as measured by horizontal photobeam breaks). Conversely, to determine activity-associated metabolic rate, we averaged values obtained from the three epochs where each mouse had the greatest activity. We performed ANCOVA analysis to determine significant differences in metabolic parameters ($\dot{V}O_2$, DO_2, O_2out, $\dot{V}CO_2$, DCO_2, CO_2out, heat generated) as a function of body adiposity [5]. Bonferroni-corrected two-tailed t-tests were used to assess for differences in remaining DEXA parameters: bone mass density (BMD), bone mineral content (BMC), bone area (BArea), tissue area (TArea), ratio of soft tissue attenuation (RSA), total tissue mass (TTM), percent adiposity, and weight.

Cohort sizes were determined by power analysis to detect >10% difference in adiposity between cohorts. Cohort sizes for the depicted experiment (Supplemental Figure 2A) were Y (n=10), M (n=9), A (n=10). A pilot experiment (with slightly smaller cohort n) yielded the same findings. All members within a cohort represented biological replicates. Investigators were not blinded to mouse age when placing mice in the calorimetry chambers; however, the automated nature of data measurement and workflow minimized potential bias. Mice were randomly assigned to the order they received DEXA scanning and the calorimetry chamber where they were placed.

RNA sample preparation

RNA was purified by well-established methods (RNAqueous and RNALater, Ambion, for tissue samples; μMACS, Miltenyi, for cell suspension samples), and underwent extensive quality control to assure that only samples meeting stringent standards for purity and minimal degradation would be tested. We used a TissueLyser II (Qiagen, Valencia CA) and a Qiacube (Qiagen) for tissue sample dissociation and RNA purification to limit variability in yield and degradation. Samples were assayed for RNA

concentration and purity (NanoDrop II, Thermo, Waltham MA) and degradation (BioAnalyzer 2100, Agilent Technologies). Samples with A260/A280 ratios less than 1.8 were unsuitable for analysis secondary to RNA contamination. Samples with larger 18S instead of 28S peaks were unsuitable for microarray analysis secondary to RNA degradation. For microarray studies, RNA labeling, hybridization, and scanning was performed per manufacturer directions (www.agilent.com/chem/dnamanuals-protocols).

Human cerebellar autopsy gifts

Gift cerebellar tissue from autopsy donations was obtained from the UNMC brain bank. Post mortem samples were collected within 24 hours of death by the Department of Neuropathology, and snap frozen in liquid nitrogen. All brain samples were confirmed by light microscopy techniques to have no ongoing neurodegenerative processes. Cause of death was obtained from medical records; we excluded any samples where prolonged hypoxemia, stroke, disseminated coagulopathy/infectious agent, or head trauma preceded death. Samples with post mortem intervals > 24 hr were also excluded, as were samples displaying significant acidosis (pH<6.2). We identified 30 cases: young (32-46 y/o; *n*=10), middle-aged (61-69 y/o; *n*=10), and aged (79-99 y/o; *n*=10). For each group, all cases were biological replicates. We obtained 1 g of tissue from each case; tissue was blocked to include both molecular and internal granule cell layers, but exclude deep white matter or cerebellar nuclear structures. Each tissue was separated into 5 aliquots of 200 mg each and immersed in RNALater. Samples were homogenized (2 min, 30 Hz, 4 °C, Tissuelyzer II, Qiagen) and RNA purified as described above. 23 samples (8 young, 7 middle-aged, 8 aged) yielded cerebellar RNA with RIN>6 (BioAnalyzer 2300, Qiagen) [6]. No mobility or locomotor phenotype information was available for subjects enrolled in the brain bank.

RT-qPCR measures of gene expression

RT-qPCR (*Taq* polymerase, SYBR green) was performed per standard protocol in 96 well plates. Master mix was prepared per manufacturer's instructions and primers (QuantiTect®, Qiagen) added at 0.5 µl/reaction. Samples were run in triplicate (Mastercycler ep *realplex*, Eppendorf). Technical replicates that did not amplify a PCR product were censored from the final dataset. Controls included (1) positive control, (2) no RT with RNA and master mix, and (3) no RNA with RT and master mix. Randomization was not required for this experiment. RT-qPCR cycle was converted to relative gene expression by the comparative C_T method [7]. MIQE checklists [8] for RT-qPCR experiments validating microarray studies and assaying microglial gene expression are provided in Supplemental Data. Sample sizes were arrived at through prior experience, and were designed such that any measure of gene expression derived from at least 5 biological replicates.

Microarrays

Resources were available to perform microarray analysis on a randomly selected subset of male BALB and C57BL/6 mice after completion of home cage behavioral studies (2-3 mo old, *n*=7, 12-13 mo old, *n*=5, 20-21 mo old, *n*=6 for BALB hypothalamus; 2-3 mo old, *n*=5, 22-24 mo old, *n*=5 for C57BL/6 hypothalamus; 2-3 mo old, *n*=5, 12-13 mo old, *n*=6, 21-23 mo old *n*=4 for BALB cerebellum; 2-3 mo old, *n*=4, 12-13 mo old, *n*=2, 21-23 mo old, *n*=6 for C57BL/6 cerebellum). Prior to arraying, samples received one round of amplification per Agilent protocol. Samples were hybridized onto Agilent Whole Mouse Genome 4x44k arrays (G4122F, 07Aug2006, 014868, www.chem.agilent.com/ cag/ bsp/ arrayspecs_final.asp). Of note, these arrays use 60-mer probes to detect 41,174 full length mouse genes and ESTs. Quality control measures (using Feature Extraction 9.1, Agilent Technologies) assessed chip performance across all samples and did not identify any outliers. We used quantile normalization [9] to adjust all signal strengths for comparison. Sample sizes were arrived at through prior experience and in consultation with the Functional Genomics Core, and were designed to array at least 5 biological replicates for all study cohorts. Cohorts may ultimately have <5 biological replicates due to technical problems precluding use of specific samples. Two technical replicates were prepared for each experiment. No significant differences were appreciated across technical replicates; we randomly chose one of these replicates for further analysis. Technologists were blinded regarding mouse cohort throughout sample preparation and array hybridization/scanning. Cost constraints prevented repeating this entire experiment; however, RT-qPCR experiments (as described above) were used to validate array results.

DEG identification

Following quality control, array data was analyzed using software packages (*limma, stat*) from the publicly available Bioconductor suite (version 1.9, running on R version 2.10, www.bioconductor.org, www.r-project.org). These packages were used to calculate B [10] and FDR statistics to identify DEGs. These

methods differ in relative stringency. The B statistic has the least stringent multiple comparison criteria, and is thus likely to identify the most DEGs. Criteria for determining differentially expressed genes are B values greater than zero, and *p* values for the FDR < 0.05.

Classification of DEGs by ontology

Following DEG identification, we used currently accepted gene ontology-based methods for initial DEG functional characterization and to determine interactions between DEGs. We employed the publically available PANTHER (www.pantherdb.org) [11] and OntoExpress (vortex.cs.wayne.edu/projects.htm) [12] tools for these analyses. Parameters for PANTHER were as follows: Tools tab, Gene Expression Data Analysis link, Compare Gene Lists link, Select organism: Mus musculus; List type: Gene, Transcript, Protein and Alternative ID; Upload list: available upon request; Reference List: Mus musculus; Search options: GO Biological processes. Parameters for OntoExpress were as follows: Annotation database: ontotools database; Organism: mus musculus; Input file: available upon request; Input type: affymextrix probe id; Reference array: agilent technologies mouse genome, whole g4122f; Distribution: hypergeometric; Correction: fdr. Both tools yielded concordant results. We also used currently available genomic resources, including Information Hyperlinked over Proteins (iHOP; www.ihop-net.org/UniPub/iHOP), and Online Mendelian Inheritance in Man (OMIM, www.ncbi.nlm.nih.gov/omim) to manually curate gene functional data.

Probability of differing CNS regions showing similar patterns of gene expression

Our statistical experts employed a bootstrapping based technique (implemented in R) to determine the probability that the C57BL/6 cerebellum and BALB hypothalamus would express the same 45 genes (as assessed by an Agilent 4x44K whole mouse genome platform) by chance (per Venn diagram, Figure 4). The R code we developed is as follows:

```
set.seed(1)
N=42000 # total number of genes
H=101 # condition 1
M=153 # condition 2
k=0
ID=NULL
for (i in 1:100000){
Y=sample (N, H, replace=F)
X=sample (N, M, replace=F)
aa=length (intersect(Y,X))
ID[i]=aa
if (aa>=45) #45 genes overlapped
k=k+1
}
Pvalue=(k/100000)*100
```

Preparation of CNS single cell suspensions

Single cell suspensions (SCSs) of the hypothalamus and cerebellum were prepared using a commercially available papain-based enzymatic digestion kit (Worthington Biochemicals, Lakewood, NJ) following the method of Huettner and Baughman [13]. Briefly, following sacrifice, CNS tissue was dissociated in solution containing Earle's Balanced Salt Solution (EBSS), papain, and EDTA, and placed in a 5% CO_2 incubator for 40 min at 37°. Tissue was thoroughly triturated into a suspension, filtered at 40 µm, and resuspended in EBSS/ albumin ovomucoid inhibitor/ DNAase. Cells were harvested from the pellet by density centrifugation. Sample sizes were arrived at by power analysis designed to detect two-fold or greater increases or decreases in gene expression. Estimates for sample mean and variance were derived from pilot experiments. Technicians and investigators were blinded to mouse cohort for SCS preparation.

Microglial enrichment

We enriched the above SCS for microglia using a magnetic-bead based approach (Miltenyi Biotec, Auburn, CA). First, SCSs were mixed with anti-myelin magnetic beads (50 µl young cohort, 75 µl middle-aged cohort, 100 µl aged cohort, 130-094-544, Miltenyi), kept at 4° for 15 min, and filtered through an automatic magnetic cell sorter (autoMACS, DEPL05 setting, Miltenyi), keeping the flow-thru and discarding the bead-bound elements. The myelin-depleted SCSs were then mixed with anti-CD11B magnetic beads (10 µl, 130-093-634, Miltenyi), kept at 4° for 15 min, and twice-filtered through the autoMACS (POSSEL setting), now discarding the flow-thru and keeping the bead-bound elements. SCSs at this stage are both myelin-depleted and CD11B (presumptive microglia) enriched. Technicians and investigators were blinded to mouse cohort for microglial enrichment.

RNA isolation from SCS

We employed magnetic bead separation to obtain mRNA from SCSs (Miltenyi). Briefly, SCSs after microglial enrichment underwent cell lysis, centrifugation, resuspension in lysis/binding buffer (high salt, 1% SDS), labeling with 50 µl oligo-dT magnetic beads (µMACS, 130-075-201, Miltenyi), and passage through a magnetic column. mRNA was then

washed with a low salt buffer and eluted into RNAase-free water. The beads capture messages possessing a 3' tail; smaller RNAs and immature or lncRNAs without a tail are discarded in the flow-thru.

Preparation of cerebellar ultrathin sections

To measure synaptic counts at highest accuracy, we employed a modified array tomography protocol [14]. Fixed whole hemicerebellum tissues were washed 3 times in 0.1M PBS, 15 min each. Samples were dehydrated in a graded ethanol series (50%, 70%, 90%, 95%, 100%, 100%, 100%) for 15 min at each step. Samples were soaked in 2 changes of LR White™ (Structure Probe Inc., West Chester PA), 15 minutes each step. Tissues were soaked overnight in LR White™. The following day, tissues were placed in flat-bottomed embedding capsules containing fresh LR White™ and oriented for sectioning. A Pelco Biowave (Ted Pella, Inc., Redding CA) equipped with the Pelco Coldspot stage and Pelco SteadyTemp was used for the initial polymerization. Capsules were submerged in water and the initial polymerization carried out per the following steps: 250 watts for 1.5 hrs to cutoff temperature of 77 °C; 250 watts for 15 min to 80.7 °C; 250 watts for 15 min to 87.4 °C; 250 watts for 10 min to 90.1 °C. After initial polymerization, the blocks were placed in the embedding oven at 65 °C and left overnight. Sections were cut 90 nm thick using a diamond knife (Histo, DiATOME Inc., Hatfield PA) on an ultramicrotome (Ultracut, Leica Microsystems, Buffalo Grove IL). Sections were heat fixed to immune treated glass slides. Sample sizes were derived from prior experience, with specific care taken to ensure at least 5 biological replicates were obtained for each cohort. Randomization was not required for this experiment. Cost and time constraints prevented replication of these findings.

Preparation of hypothalamic thin sections

Whole brains were cryoprotected following serial overnight immersion in 4% PFA. The next day, brains were rinsed in 20% sucrose followed by overnight immersion in 20% sucrose in 0.1M PBS. The following day, brains were transferred to fresh 20% sucrose in 0.1M PBS and kept at 4 °C for three days. Brains were flash frozen by slow immersion in isopentane on dry ice. Frozen tissue was stored at -80 °C until processing. Tissue was embedded in OCT Compound (4583, Sakura), and coronal 10 µm thick sections were cut on a cryostat (CM1850, Leica Microsystems; Buffalo Grove IL) between bregma -1.46 and -2.51. Sections (three per slide) were thaw-mounted onto glass slides (3800092 Blue, X-tra Thermal slides, Leica Microsystems) for immunostaining.

Immunocytochemistry for ultrathin section Vglut1, C3

Slides were placed in slide holders and submerged into PBS/10% fetal bovine serum for 1 hr. After removing slides from chamber, a circle was drawn around the area containing tissue with a PAP pen (DAKO, Carpinteria CA) to keep antibody solution on tissue area. Primary antibodies were directed against Vglut1 (cerebellum), and C3. All antibodies were diluted in PBS/1% rabbit serum to the following concentrations: Vglut1 (polyclonal, host guinea pig, Millipore, #AB5905) 1:4000; C3 (polyclonal, host goat, MP Biomedicals, #55444), 1:400. We placed 50-60 µl of the diluted antibody mix to completely cover the tissue. Sections were incubated at room temp for 1 hr, then washed in fresh PBS 3 times for 10 min each. Secondary antibodies were diluted 1:250 in PBS/1% rabbit serum: AlexaFluor 555 goat anti-guinea pig IgG (H+L) (Invitrogen, #A21435), AlexaFluor 488 rabbit anti-goat IgG (H+L) (Invitrogen, #A11078). We used 50-60 µl of diluted secondary antibody mix to completely cover tissue. Sections were incubated at room temperature in the dark for 1 hour. Sections were washed in fresh PBS three times for 10 min each. 50-60 µl 4’,6-diamidino-2-phenylindole (DAPI, Invitrogen) was added to each slide for 1 min. Slides were rinsed in PBS for 5 min, and then fixed using Prolong Gold Antifade Reagent (Invitrogen P36934). We placed the cover slip over the tissue and sealed all edges with nail polish. Slides were dried in a dark area for 30-45 min. Slides were stored at 4 °C short term or -80 °C long term.

Immunochemistry for thin section Vglut1, C3

Slides were equilibrated to room temp for 30 min and then rinsed 3x with PBS for 10 min each. A circle was made around each tissue section with a PAP pen. 50 µl of 5% BSA plus 0.2% Triton X in PBS were applied to each tissue. Slides were then placed in a humidity chamber for 60 min. Primary antibody was diluted in 0.5% BSA and 0.05% Triton X in PBS at the following ratios: VGlut1 (Millipore, #AB5905) 1:4000, and C3 (clone 11H9, Abcam, Ab11862) 1:300. We placed 50-60 µl of diluted antibodies to completely cover the tissue. Slides were incubated at 4 °C overnight. The following day, slides were washed in fresh PBS in chamber 3 times for 10 min each. Secondary antibody was diluted in 0.5% BSA and 0.05% Triton X in PBS at the following ratios: AlexaFluor 488 goat anti-guinea pig IgG (H+L) (Invitrogen, #A11073) 1:250, and AlexaFluor 555 goat anti-rat IgG (H+L) (Invitrogen,

#A21434) 1:250. 50-60 µl of diluted antibodies were pipetted to completely cover tissue. Slides were incubated at room temperature in the dark for 2 hours. Following secondary antibody incubation, slides were washed in fresh PBS 3 times for 10 min each. DAPI staining, slide fixing, coverslipping, and storage as described above.

Immunocytochemical quantification

When imaging the cerebellum, we evaluated 6-13 nonoverlapping windows fully containing histologically identified internal granule cell layer. Specifically, for the BALB internal granule cell layer, we evaluated 34 windows from three 2-3 mo old mice and 32 windows from three 21-24 mo old mice. For the C57B/L6 internal granule cell layer, we evaluated 38 windows from four 2-3 mo old mice and 42 windows from four 21-24 mo old mice. Unless otherwise mentioned, we were able to simultaneously evaluate Vglut1 and C3 expression from the same slides. All cerebellar images were obtained on a Zeiss 710 confocal microscope (Oberkochen, Germany) under 40x objective with 4x zoom. When imaging the hypothalamus, we evaluated 4-6 nonoverlapping windows from sections at approximate bregma -1.49, -1.58, and -1.67. For the BALB arcuate nucleus we evaluated 81 windows from seven 2-3 mo old mice and 86 windows from seven 21-24 mo old mice (both C3 and Vglut1); for the C57BL/6 arcuate nucleus, we evaluated 57 windows from four 2-3 mo old mice and 70 windows from five 21-24 mo old mice. We simultaneously evaluated Vglut1 and C3 expression from the same slides. Standard atlas was used to determine position and approximate boundaries of the hypothalamic nuclei. All hypothalamic images were obtained on a Zeiss 710 confocal microscope under 40x objective/4x digital zoom (420462-9900 EC Plan Neofluar, Zeiss) with 1,3 oil DIC.

We used ImageJ to perform unbiased stereological analysis per accepted standards. In addition to the automated nature of this workflow, investigators were further blinded to mouse age when performing these quantifications. Images were exported as three-color TIF files. The global scale for the image windows taken at 40x with 4x zoom was set according to the objective, zoom, and resolution at 0.039 µm for cerebellar images and 0.052 µm for hypothalamus images. For each starting image (Supplemental Figure 5A), the rolling ball radius of the subtract background function was set to a value several times larger than the puncta of interest to visually evoke an adequate decrease in background signal (Supplemental Figure 5B). Each image was split into an RGB stack (Supplemental Figure 5C). The background subtraction value was set for each batch of images (*i.e*. Young and Old Cerebellum Vglut). Next, the threshold was set for each batch of images visually where the fainter puncta were quantifiable while the brighter puncta remained distinct from surrounding puncta (Supplemental Figure 5D). The smooth function was used to blur the puncta boundaries (Supplemental Figure 5E), and convert the image to a binary format (Supplemental Figure 5F). To separate closely positioned puncta, the watershed function was used (Supplemental Figure 5G), adding a 1-pixel thick line between areas of apparent separation. Quantification was achieved using the Analyze Particles function controlling for particle size and circularity (Supplemental Figure 5H). To assess the accuracy of the counts visually, the masks feature was used (Supplemental Figure 5I). The number of puncta, total area of puncta, and average puncta size (μm^2) were recorded for each image. Total count data (Vglut1, C3) was analyzed by 2-way ANOVA, with age and strain as primary factors, immunocytochemical processing run and subject number as covariates. Counts from the internal granule cell layer and arcuate nucleus were analyzed in separate 2-way ANOVAs. When required, *post hoc* testing between groups was by Bonferroni method.

Manganese-enhanced MRI

Mice of strain BALB (5 young, 5 aged) and C57BL/6 (5 young, 7 aged) underwent MRI scanning before and after 4 daily *i.p.* injections of $MnCl_2$ (50 mM solution to dosage 60 mg/kg, daily *ip* injection). Mice were anesthetized with isoflurane (0.5 to 1.5 volume % to maintain respirations between 40-80/min) and laid on their stomach in a nonmagnetic holder with their heads secured by ear bars. Mice were scanned in a 7 T, 21 cm MRI system (Bruker, Billerica MA) with a custom surface coil as RF receiver. Coil characteristics have been previously published [15]. Sample sizes were arrived at by both prior experience and in consultation with the Small Animal Imaging Core. Cost constraints made it unfeasible to replicate this experiment.

T_1 mapping by progressive saturation was performed using a fast spin echo sequence with variable TRs from 480-9000 ms, 10 dummy pulses, RARE factor 2, TE 9.8, 19.6 ms, nominal TE = 9.8 ms, slice thickness 0.5 mm, 24 slices, matrix 176 x 156 in plane with 100 µm x 100 µm isotropic in-plane pixel size. Three-dimensional T_1-weighted MRI was acquired using a gradient echo sequence with TR/TE 20/4.5 ms, averages 2, flip angle 20°, matrix 176 x 128 x 128, isotropic pixel size 100 µm.

Diffusion tensor images (DTI) were acquired using a 3D echo planar imaging sequence, 2 shot acquisition, with a volume coil transmit, four channel surface coil

receive, GRAPPA acceleration factor = 2, TE = 19.5 ms, TR = 1000 ms, acquisition matrix of 164 x 164 x 24, nominal slice thickness = 0.5 mm, zero filled to 256 x 256. Diffusion encoding was done using a balanced, rotationally invariant and alternating polarity icosahedral scheme (12 directions) designed to reduce background-diffusion gradient coupling [16, 17]. Diffusion weighting b-factor = 800 s/mm, δ=4 ms, Δ=15 ms, G_{dmax} = 40 G/cm, 200 ms rise time, 4 averages for b=0 acquisition, 5 averages for each b=800 encoding direction, for a total acquisition time of 51 minutes, 12 seconds.

Image analysis

Throughout all stages of image analysis, investigators were blinded to age. Images underwent brain extraction using DefCon/BSE_LS44 [18] followed by inhomogeneity N3 shading correction [19] (as implemented on MIPAV [20]). Whole brain signal intensity (SI) was normalized to mean SI across all mice to minimize inter- and intra-subject scan variability. Brain extracted images of pre- and post-$MnCl_2$ administration were first registered in a pairwise manner using rigid body transformation, followed by affine transformation methods. To minimize interpolation errors, transformation matrices were combined together and applied in one step to original pre-$MnCl_2$ data. Registrations were performed using Diffeomap 1.6v as implemented in DTIStudio software (www.mristudio.org). The SI difference (SI_d) was determined by subtracting the pre-$MnCl_2$ image from the post-$MnCl_2$ image.

Region of interest (ROI) analysis was performed on the hypothalamus and cerebellar internal granule cell layer to quantify changes in presumptive excitatory neurotransmission in these CNS regions. Using ImageJ, ROIs were overlaid on T_1-weighted images as referenced to bregma. Image intensity was subsequently measured by ImageJ [21]. ROI analysis was performed in triplicate for each region across three different levels of bregma. The same ROIs were used in all brains. ROI measurements were performed for both pre- and post-$MnCl_2$ images. These measurements were then averaged (across the three replications); averaged post-$MnCl_2$ T_1-weighted intensity values were subtracted from averaged pre-$MnCl_2$ T_1-weighted intensity values to determine $R_{(s-1)}$, a direct measure relating $MnCl_2$ uptake to changes in signal intensity.

Analyses of the diffusion-weighted data were performed using custom programs written in IDL as previously described [22-24]. Analyses produced maps of the tensor diffusivities (λ_1, λ_2, λ_3), mean diffusivity (D_{av}) where: D_{av} = 1/3*(λ_1 + λ_2 + λ_3) and fractional anisotropy (FA), where:

$$FA = \frac{1}{\sqrt{2}} \sqrt{\frac{(\lambda_1 - \lambda_2)^2 + (\lambda_2 - \lambda_3)^2 + (\lambda_1 - \lambda_3)^2}{\lambda_1^2 + \lambda_2^2 + \lambda_3^2}} .$$

Transverse ($\lambda_\perp = (\lambda_2 + \lambda_3)/2$) and longitudinal ($\lambda_\parallel = \lambda_1$) components of the diffusion tensor were obtained as previously described [25].

TABLES

Please browse the links in Full Text version of this manuscript to see Supplemental Tables:

Supplemental Table 1. Differential home cage behaviors between young and aged C57BL/6 and BALB mice. (**A**) Young *vs.* aged C57BL/6 mice. (**B**) Young *vs.* aged BALB mice. In these spreadsheets, the first column refers our abbreviation for specific behavior tested. The second column contains a brief description of this behavior. The index value is an internal reference to aid in maintaining the software package. Two columns depict mean values of each specific behavior (over entire experiment) for the young and aged cohorts. Columns headed by M#### correspond to individual mean values (over entire experiment) for each tested mouse. The final columns contain the calculated statistics (MW = Mann-Whitney nonparametric test, ttest = Student's t-test, B = fold change) and their associated p values after familywise error rate correction using FDR.

Supplemental Table 2. Differential gene expression between young and aged animals, all cohorts, all CNS regions. (**A**) Whole BALB hypothalamus. Expanded table from Figure 4 showing how specific differentially expressed loci were assigned to specific ontologies. Loci common between the BALB hypothalamus and C57BL/6 cerebellum highlighted in red. P value calculated by hypergeometric statistic and corrected for multiple comparisons across all ontologies. PANTHER database build 6 Jan 2009. (**B**) Whole BALB hypothalamus. Complete rank-ordered (by B statistic) DEG list comparing young and aged mouse cohorts. These results are mostly in agreement with past studies [26]; differences between our results and other findings may be attributable to our use of individual rather than pooled samples [27], microarray platform [27-29], and improved annotation datasets. (**C**) Whole BALB cerebellum. Table depicting significant ontologies. P value calculated by hypergeometric statistic and corrected for multiple comparisons across

all ontologies. Downgoing arrows indicated overall decreased expression of gene loci within the given ontology; upgoing arrows indicated overall increased expression of gene loci within the given ontology; filled circles indicate no change. PANTHER database build 6 Jan 2009. (**D**) Whole BALB cerebellum. Expanded table from (**C**) showing how specific differentially expressed loci were assigned to specific ontologies. (**E**) Whole BALB cerebellum. Complete rank ordered (by FDR<0.05 and absolute fold change≥3) DEG list comparing young (n=5) and aged (n=4) mouse cohorts. 985 loci identified overall. (**F**) Whole C57BL/6 hypothalamus. Expanded table showing how specific differentially expressed loci were assigned to specific ontologies. DEGs determined by FDR statistics. P value calculated by hypergeometric statistic and corrected for multiple comparisons across all ontologies. PANTHER database build 6 Jan 2009. (**G**) Whole C57BL/6 hypothalamus. Complete rank-ordered (by raw P value) DEG list comparing young and aged mouse cohorts. (**H**) Whole C57BL/6 cerebellum. Expanded table from Figure 4 showing how specific differentially expressed loci were assigned to specific ontologies. Loci common between the C57BL/6 cerebellum and BALB hypothalamus highlighted in red. P value calculated by hypergeometric statistic and corrected for multiple comparisons across all ontologies. PANTHER database build 6 Jan 2009. (**I**) Whole C57BL/6 cerebellum. Complete rank-ordered (by B statistic) DEG list comparing young and aged mouse cohorts.

FIGURES

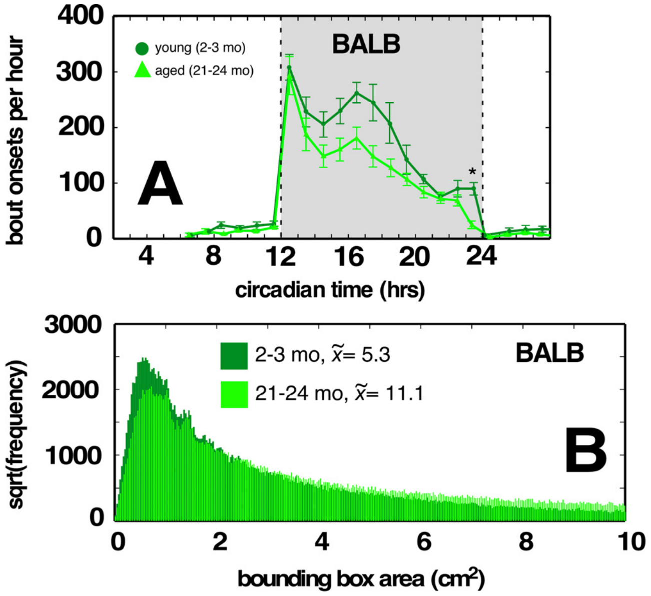

Supplemental Figure 1. Aged BALB mice have no phenotypic differences in movement bout onsets, bout speeds, or locomotor path straightness compared to young BALB mice. (**A**) No mobility impairment in aged BALB mice. (**B**) Distribution of MBR areas for young and aged BALB mice. While both genotype and across-mouse variability were significantly different between young and aged mice (p<<0.001), both cohorts showed overall MBR areas similar to young C57BL/6 mice. Traces in light green correspond to aged mice, dark green correspond to young mice. Greyed region depicts dark cycle, dashed lines indicate dark cycle onset and offset, respectively. Asterisks indicate p<0.01, Bonferroni corrected; error bars are ± 1 standard error of the mean. Distribution of minimum bounding rectangle areas (MBRs; cut off at 10 to better show small rectangle areas) for locomotor bouts of young (dark blue) and aged (red) C57BL/6 mice. Smaller MBRs indicate more direct locomotor paths. Median values for each cohort in legend.

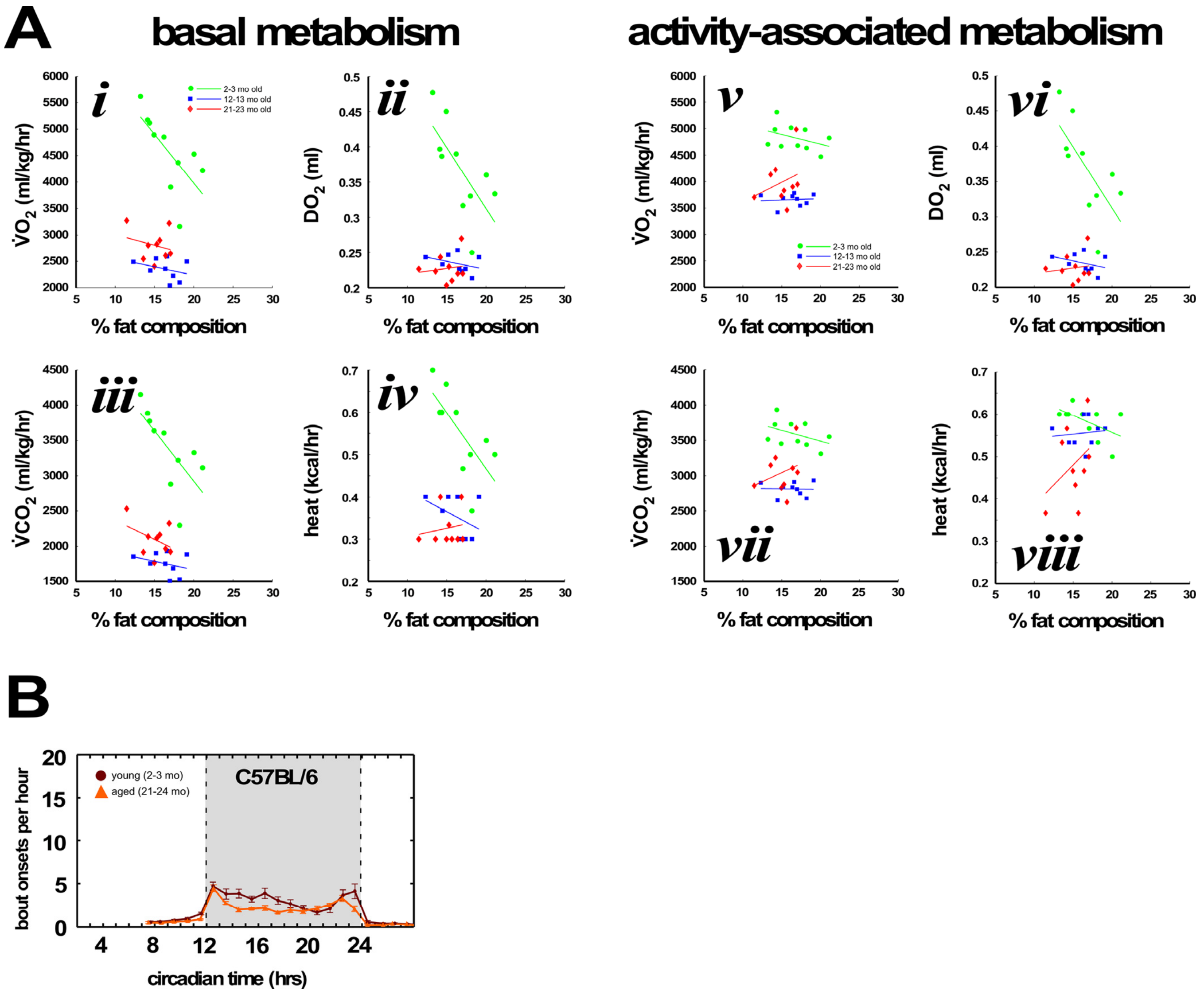

Supplemental Figure 2. Aged BALB mice have similar metabolism compared to middle-aged cohorts. (**A**) Young BALB mice (n=10) have greater peak oxygen consumption, oxygen tissue delivery, CO_2 production, and estimated heat compared to middle-aged (n=9) and aged (n=10) cohorts under both basal and activity-associated conditions. For basal $\dot{V}O_2$ (panel *i*), the primary factors age (p<<0.001) and percent adiposity (p<0.004) were significant by ANCOVA; the age by adiposity one-way interaction did not attain statistical significance. For basal DO_2 (panel *ii*), the primary factors age (p<<0.001) and percent adiposity (p<0.006), and the age by adiposity one-way interaction (p<0.01) all were significant by ANCOVA. For basal $\dot{V}CO_2$ (panel *iii*), the primary factors age (p<<0.001) and percent adiposity (p<0.003) were all significant by ANCOVA; the age by adiposity one-way interaction was not. For basal heat production (panel *iv*), the primary factors age (p<<0.001) and percent adiposity (p<0.009) were all significant by ANCOVA; the age by adiposity one-way interaction was not. For activity-associated $\dot{V}O_2$ (panel *v*), the primary factor age (p<<0.001) was significant by ANCOVA; the primary factor percent adiposity and the age by adiposity one-way interaction did not attain statistical significance. For activity-associated DO_2 (panel *vi*), the primary factors age (p<<0.001) and percent adiposity (p<0.007), and the age by adiposity one-way interaction (p<0.04) were all significant by ANCOVA. For activity-associated $\dot{V}CO_2$ (panel *vii*), the primary factor age (p<<0.001) was significant by ANCOVA; the primary factor percent adiposity and the age by adiposity one-way interaction did not attain statistical significance. Finally, for activity-associated heat production (panel *viii*), the primary factor age (p<0.004) was significant by ANCOVA; the primary factor percent adiposity and the age by adiposity one-way interaction did not attain statistical significance. (**B**) No feeding impairment in aged C57BL/6 mice. Traces in light orange correspond to young mice; traces in dark orange correspond to aged mice. Grayed region depicts dark cycle, dashed lines indicate dark cycle onset and offset, respectively. Asterisks indicate p<0.01, Bonferroni corrected; error bars are ± 1 standard error of the mean.

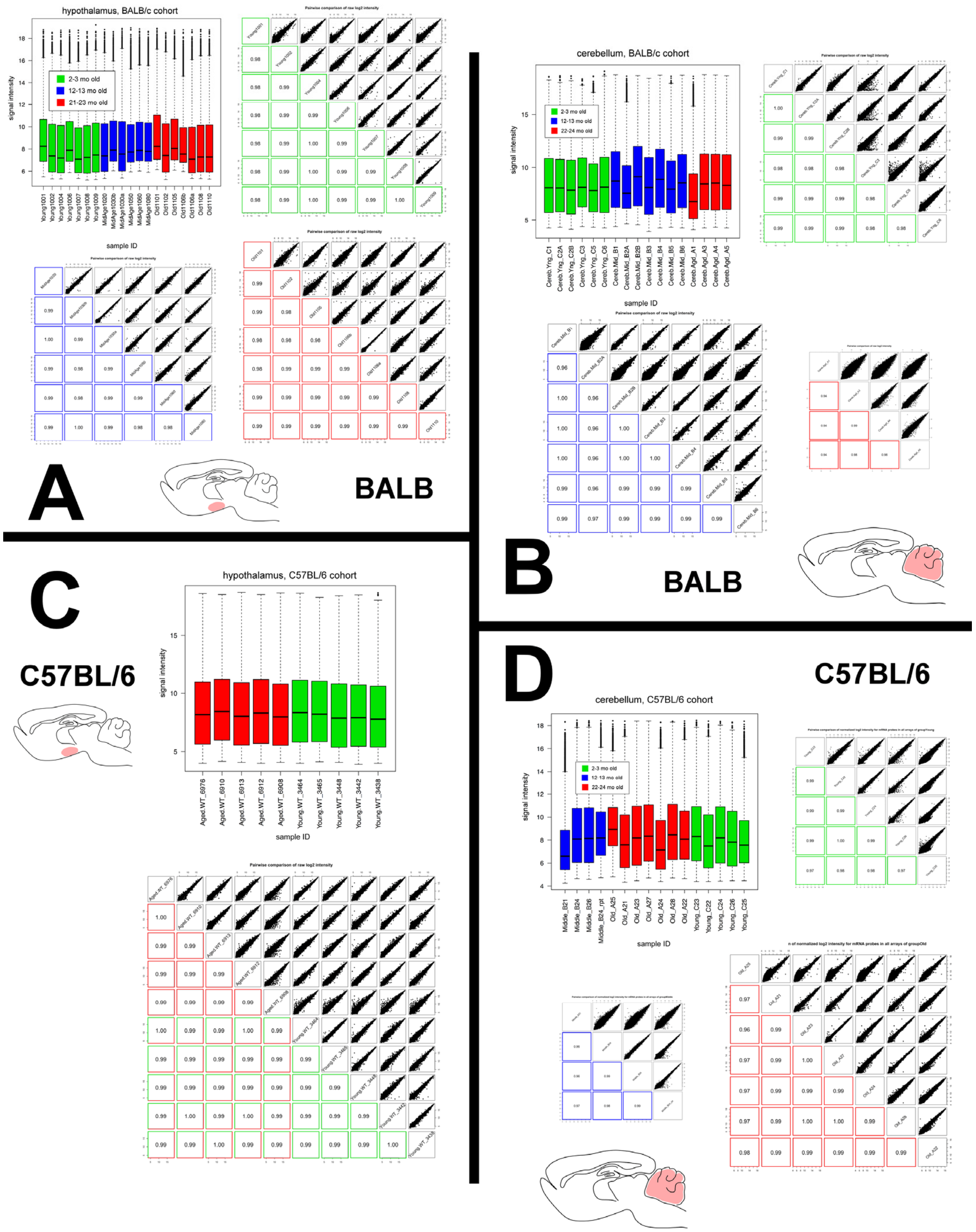

Supplemental Figure 3. Microarray quality control assessment. For all microarray quality control figures, green depicts samples from young cohorts, blue depicts samples from middle-aged cohorts, and red depicts samples from aged cohorts. Samples ending with 'a' and 'b' represent technical replicates, otherwise, samples within each group are biological replicates. (**A**) BALB hypothalamus cohorts. Upper left hand panel depicts box/whisker plot with outliers of signal intensity across the 20 chips. Each microarray array performed in a similar manner. Upper right hand panel depicts pairwise comparisons of signal strength for the young mouse cohort. Values below the diagonal depict correlations between specific samples; for example, the value of 0.98 in the lower left corner box is the pairwise signal strength correlation between young mouse 1001 and young mouse 1009. Figures above the diagonal are the respective scatter plots. Lower left hand panel depicts pairwise comparisons of signal strength for the middle-aged mouse cohort. Interpretation as above. Lower right hand panel depicts pairwise comparisons of signal strength for the aged mouse cohort. Interpretation as above. (**B**) BALB cerebellum cohorts. Interpretation identical to panel **A.** (**C**) C57BL/6 hypothalamus cohorts. Lower panel again depicts pairwise comparisons of signal strength between specific samples; for this figure, comparisons outlined in red are between two aged animals, comparisons outlined in green are between two young animals, and comparisons outlined in both red and green are between a young and aged animal. (**D**) C57BL/6 cerebellum cohorts. Interpretation identical to panel (**A**).

Biological Process	Observed	Expected	*p*
Signal transduction	203	89.18	$<1.0 \times 10^{-6}$
Development	132	47.59	$<1.0 \times 10^{-6}$
Neuronal activities	74	16.78	$<1.0 \times 10^{-6}$

BALB

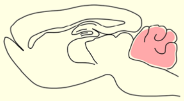

Biological Process	Observed	Expected	*p*
Signal transduction	26	13.29	1.35×10^{-2}
Development	17	7.10	1.90×10^{-2}
Neuronal activities	10	2.50	6.41×10^{-3}

C57BL/6

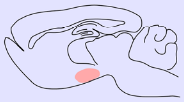

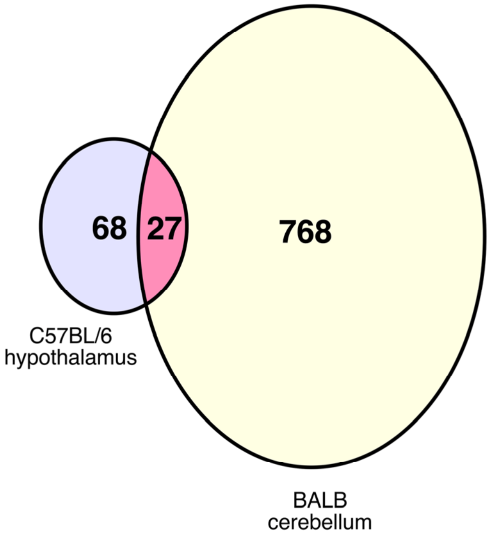

Supplemental Figure 4. Age-associated increase in C57BL/6 hypothalamic and BALB cerebellar expression of transcripts associated with signal transduction, development, and neuronal activities. Venn diagram demonstrates moderate overlap in age-related changes in gene expression. In male C57BL/6 hypothalamus, we identify 95 differentially expressed genes (DEGs), 79 upregulated in aged mice. In male BALB cerebellum, we identify 795 DEGs, 388 upregulated in aged mice. Of note, 27 of these genes are differentially expressed in both C57BL/6 hypothalamus and BALB cerebellum; the probability of this occurring by chance is $p<<0.0001$.

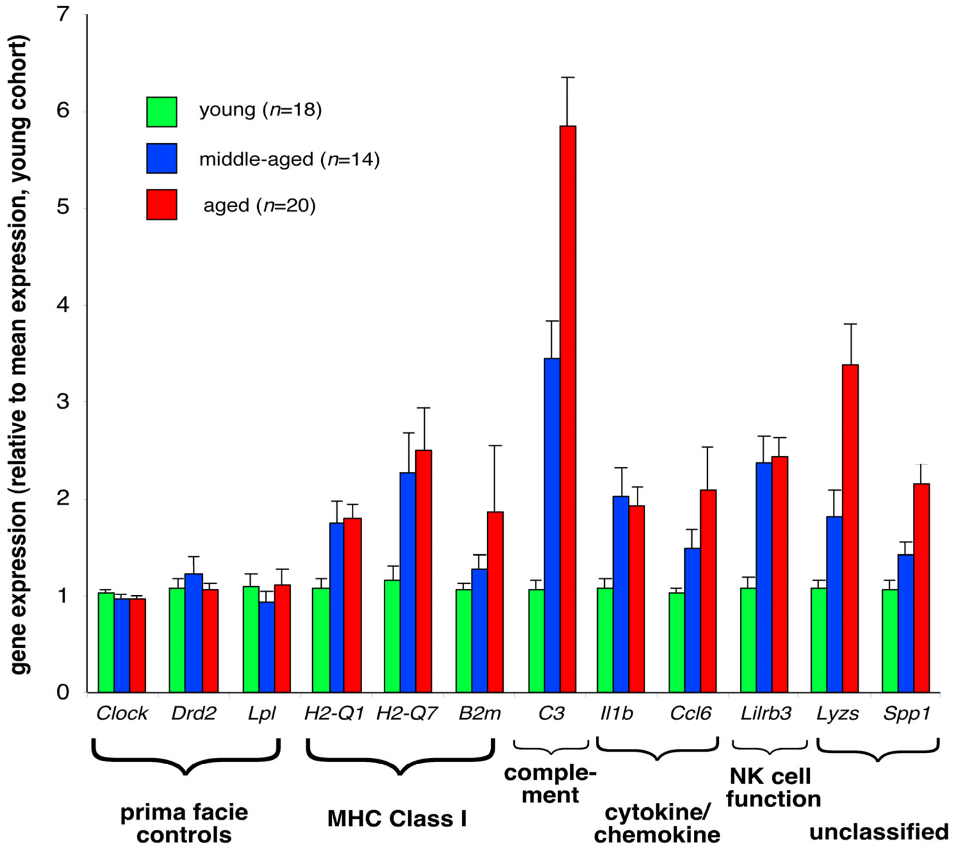

Supplemental Figure 5. RT-qPCR confirmation of age-related whole tissue hypothalamic differential gene expression observed in male BALB cohort. Mean ± 1 standard error. *Clock*, *Drd2*, *Lpl* were assayed as control genes not differentially expressed per microarray. Loci values normalized to young cohort *Gusb* expression.

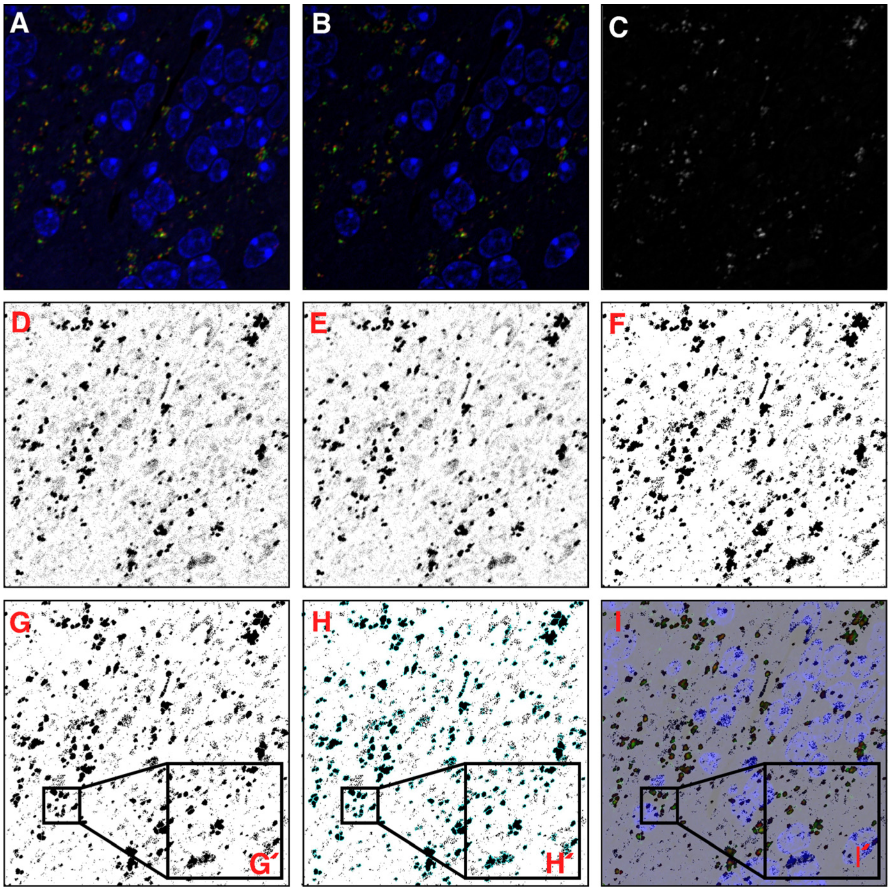

Supplemental Figure 6. Workflow for image quantification using ImageJ. We provide representative images for each step leading to puncta counts. Confocal images exported as three color TIF files and loaded into ImageJ. (**A**) Background subtraction using the subtract background function in ImageJ with a rolling ball radius of 50 pixels. (**B**) The three color image was split into an RGB stack and the channel of interest selected. (**C**) Numerous images from each batch were analyzed to find an adequate threshold where faint puncta were visible, but brighter puncta remained as distinct as possible. (**D**) The smooth function was used to blur edges of each puncta to allow for future particle analysis. (**E**) The images were converted to a binary image (**F**) and then the watershed function was used to separate closely positioned puncta (**G**, **G´**). The analyze particle feature was used, controlling for puncta size and circularity with an output of overlay outlines (**H**, **H´**). To assess the visual accuracy of the counts, the outline overlay is pasted as a blended image over the three color image with increased brightness (**I**, **I´**).

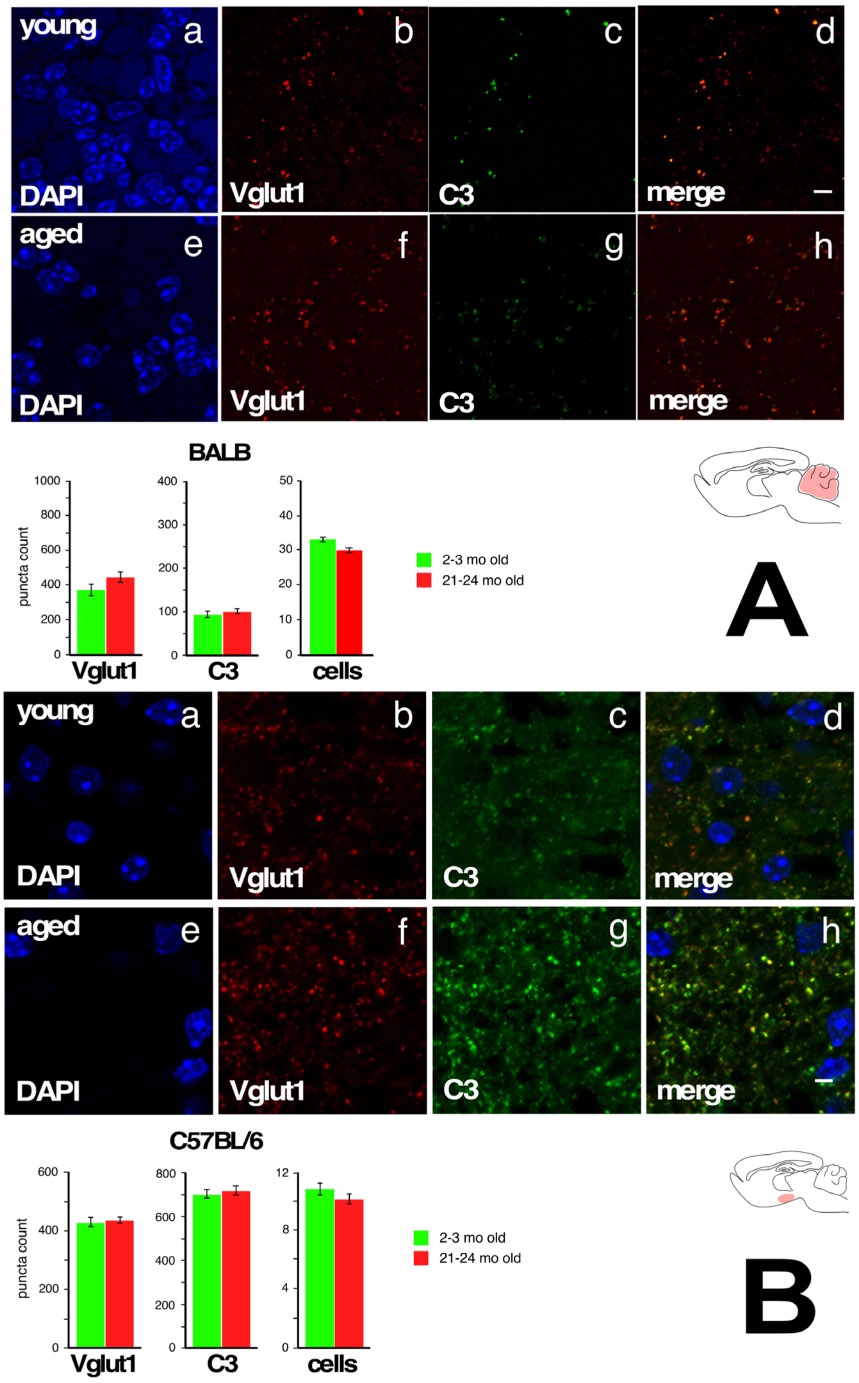

Supplemental Figure 7. No age-related changes in either Vglut1 or C3 expression in the cerebellar internal granule cell layer of BALB mice or the hypothalamic arcuate nucleus of C57BL/6 mice. (**A**) BALB cerebellar internal granule cell layer. **a**. DAPI stain, young. **b** Vglut1 immunoreactivity, young. **c**. C3 immunoreactivity, young cohort. **d** Merge, young. Note that there is significant colocalization of Vglut1 and C3 staining, particularly for more intense puncta. **e**. DAPI, aged. **f** Vglut1, aged. **g** C3, aged. **h** Merge, aged. **i** Quantification of Vglut1, C3, and DAPI. (**B**) C57BL/6 hypothalamic arcuate nucleus. Panels **a**-**h** as above. Bars are ± 1 standard error of the mean. Scale bar 4 μm.

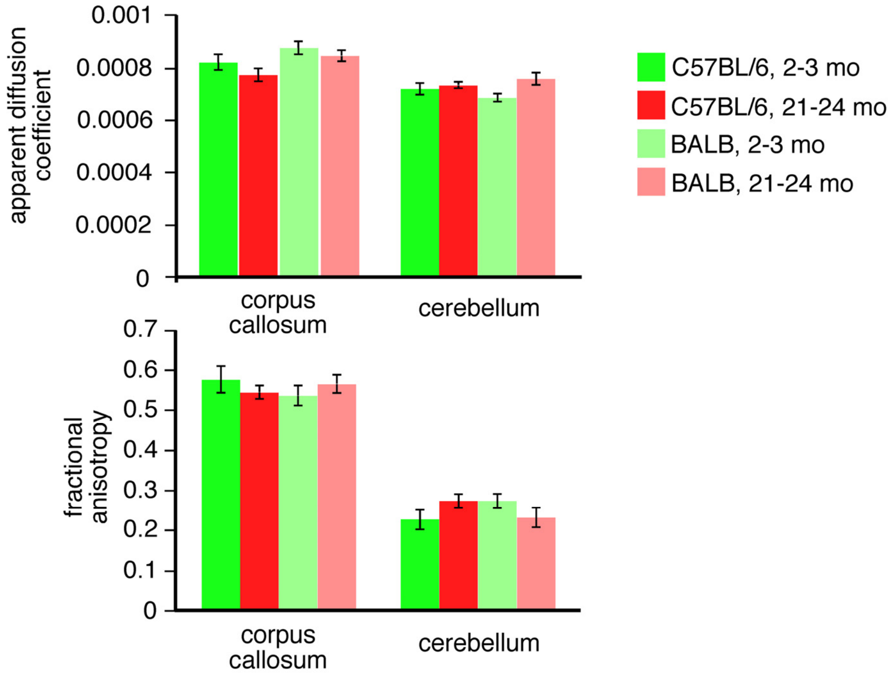

Supplemental Figure 8. No evidence of age-related vascular disease in either C57BL/6 or BALB mice as assessed by diffusion tensor imaging (DTI). Top panel: apparent diffusion coefficient, a measure of the freedom water has to diffuse within a given voxel. Left group shows values for corpus callosum, the region with the highest white matter signal within the CNS; the right group shows values for the cerebellum. No significant differences by either age or strain appreciated. Bottom panel: fractional anisotropy, a measure of the difference between the diffusion tensor long and short axes (dictated by cell geometry) within a given voxel. Panel layout as above. Again, no significant differences by age or strain appreciated.

REFERENCES

1. Goulding EH, Schenk AK, Juneja P, MacKay AW, Wade JM, Tecott LH. A robust automated system elucidates mouse home cage behavioral structure. PNAS USA. 2008; 105:20575-82.

2. Parkison S, Carlson JD, Chaudoin TR, Hoke TA, Schenk AK, Goulding EH, Pérez LC, Bonasera SJ. A low cost, reliable, high-throughput system for rodent behavioral phenotyping in a home cage environment. In Engineering in Medicine and Biology Society (EMBC). Annual International Conference of the IEEE. 2012: 2392-95, 2012, IEEE

3. Rosin PL. Measuring rectangularity. Machine Vision Appl. 1999; 11:191-196.

4. Benjamini Y, Hochberg Y. Controlling the false discovery rate: a practical and powerful approach to multiple testing. J Royal Stat Soc Series B (Methodological) 1995; 289-300.

5. Tschöp MH, Speakman JR, Arch JR, Auwerx J, Brüning JC, Chan L, Eckel RH, Farese Jr RV, Galgani JE, Hambly C, Herman MA. A guide to analysis of mouse energy metabolism. Nature Methods. 2012; 9:57–63.

6. Schroeder A, Mueller O, Stocker S, Salowsky R, Leiber M, Gassmann M, Lightfoot S, Menzel W, Granzow M, Ragg T. The RIN: an RNA integrity number for assigning integrity values to RNA measurements. BMC Mol Biol. 2006; 7:3.

7. Livak KJ, Schmittgen TD. Analysis of relative gene expression data using real-time quantitative PCR and the 2(-Delta Delta C(T)) Method. Methods. 2001; 25:402-408.

8. Bustin SA, Benes V, Garson JA, Hellemans J, Huggett J, Kubista M, Mueller R, Nolan T, Pfaffl MW, Shipley GL, Vandesompele J. The MIQE Guidelines: Minimum Information for Publication of

Quantitative Real-Time PCR Experiments. Clin Chem. 2009; 55:611-22.

9. Bolstad BM, Irizarry RA, Åstrand M, Speed TP. A comparison of normalization methods for high density oligonucleotide array data based on variance and bias. Bioinformatics. 2003; 19:185-93.

10. Lönnstedt I, Speed TP. Replicated microarray data. Statistica sinica. 2002; 12:31-46.

11. Thomas PD, Campbell MJ, Kejariwal A, Mi H, Karlak B, Daverman R, Diemer K, Muruganujan A, Narechania A. PANTHER: a library of protein families and subfamilies indexed by function. Genome Res. 2003; 13:2129-41.

12. Draghici S, Khatri P, Bhavsar P, Shah A, Krawetz SA, Tainsky MA. Onto-tools, the toolkit of the modern biologist: onto-express, onto-compare, onto-design and onto-translate. Nucleic Acids Res. 2003; 31:3775-81.

13. Huettner JE, Baughman RW. Primary culture of identified neurons from the visual cortex of postnatal rats. J Neurosci. 1986; 6:3044-60.

14. Micheva KD, Smith SJ. Array tomography: a new tool for imaging the molecular architecture and ultrastructure of neural circuits. Neuron. 2007; 55:25-36.

15. Bade AN, Zhou B, Epstein AA, Gorantla S, Poluektova LY, Luo J, Gendelman HE, Boska MD, Liu Y. Improved visualization of neuronal injury following glial activation by manganese enhanced MRI. J Neuroimmune Pharmacol. 2013; 8:1027-36.

16. Neeman M, Freyer JP, Sillerud LO. A simple method for obtaining cross-term-free images for diffusion anisotropy studies in NMR microimaging. Mag Res Med. 1991; 21:138-43.

17. Neeman M, Jarrett KA, Sillerud LO, Freyer JP. Self-diffusion of water in multicellular spheroids measured by magnetic resonance microimaging. Cancer Res. 1991; 51:4072-79.

18. Uberti MG, Boska MD, Liu Y. A semi-automatic image segmentation method for extraction of brain volume from in vivo mouse head magnetic resonance imaging using Constraint Level Sets. J Neurosci Methods. 2009; 179:38.

19. Sled JG, Zijdenbos AP, Evans AC. A nonparametric method for automatic correction of intensity nonuniformity in MRI data. IEEE Trans Medical Imaging. 1998; 17:87-97.

20. McAuliffe MJ, Lalonde FM, McGarry D, Gandler W, Csaky K, Trus BL. Medical image processing, analysis and visualization in clinical research. In Computer-Based Medical Systems, 2001. CBMS 2001. Proceedings. 14th IEEE Symposium. 2001; 381-86. IEEE.

21. Schneider CA, Rasband WS, Eliceiri KW. NIH Image to ImageJ: 25 years of image analysis. Nature Methods. 2012; 9:671-75.

22. Basser PJ, Mattiello J, LeBihan D. Estimation of the effective self-diffusion tensor from the NMR spin echo. J Magn Reson Series B. 1994; 103:247-54.

23. Basser PJ, Mattiello J, LeBihan D. MR diffusion tensor spectroscopy and imaging. Biophys J. 1994; 66:259-67.

24. Hasan KM, Basser PJ, Parker DL, Alexander AL. Analytical computation of the eigenvalues and eigenvectors in DT-MRI. J Magn Reson. 2001; 152:41-47.

25. Hasan KM. Diffusion tensor eigenvalues or both mean diffusivity and fractional anisotropy are required in quantitative clinical diffusion tensor MR reports: fractional anisotropy alone is not sufficient. Radiology. 2006; 239:611-612; author reply 2-3.

26. Prolla TA. DNA microarray analysis of the aging brain. Chemical Senses. 2002; 27:299-306.

27. Jiang CH, Tsien JZ, Schultz PG, Hu Y. The effects of aging on gene expression in the hypothalamus and cortex of mice. PNAS USA. 2001; 98:1930-34.

28. Zahn JM, Poosala S, Owen AB, Ingram DK, Lustig A, Carter A, Weeraratna AT, Taub DD, Gorospe M, Mazan-Mamczarz K, Lakatta EG. AGEMAP: a gene expression database for aging in mice. PLoS Genetics. 2007; 3:e201.

29. Xu X, Zhan M, Duan W, Prabhu V, Brenneman R, Wood W, Firman J, Li H, Zhang P, Ibe C, Zonderman AB. Gene expression atlas of the mouse central nervous system: impact and interactions of age, energy intake and gender. Genome Biol. 2007; 8:R234.

MIQE, GEO, UNMC

Please browse link in Full Text version of this manuscript to see Minimum information to describe RT-qPCR experiments.

GEO accession number: GSE87102: http://www.ncbi.nlm.nih.gov/geo/query/acc.cgi?acc=GSE87102

UNMC digital link to the raw behavioral dataset: http://digitalcommons.unmc.edu/geriatrics_data/2/.
